# Efficacy, effectiveness, and safety of herpes zoster vaccines in adults aged 50 and older: systematic review and network meta-analysis

**DOI:** 10.1136/bmj.k4029

**Published:** 2018-10-25

**Authors:** Andrea C Tricco, Wasifa Zarin, Roberta Cardoso, Areti-Angeliki Veroniki, Paul A Khan, Vera Nincic, Marco Ghassemi, Rachel Warren, Jane P Sharpe, Andrea V Page, Sharon E Straus

**Affiliations:** 1 Knowledge Translation Program, Li Ka Shing Knowledge Institute, St Michael’s Hospital, 209 Victoria Street, East Building, Toronto, M5B 1W8, ON, Canada; 2 Epidemiology Division, Dalla Lana School of Public Health, University of Toronto, 155 College Street, Toronto, M5T 3M7, ON, Canada; 3 Division of Infectious Diseases, Mount Sinai Hospital, Joseph and Wolf Lebovic Health Complex, Toronto, ON, Canada; 4 Department of Medicine, University of Toronto, Toronto, ON, Canada; 5 St Michael’s Hospital, Toronto, ON, Canada

## Abstract

**Objective:**

To compare the efficacy, effectiveness, and safety of the herpes zoster live attenuated vaccine with the herpes zoster adjuvant recombinant subunit vaccine or placebo for adults aged 50 and older.

**Design:**

Systematic review with bayesian meta-analysis and network meta-analysis.

**Data sources:**

Medline, Embase, and Cochrane Library (inception to January 2017), grey literature, and reference lists of included studies.

**Eligibility criteria for study selection:**

Experimental, quasi-experimental, and observational studies that compared the live attenuated vaccine with the adjuvant recombinant subunit vaccine, placebo, or no vaccine in adults aged 50 and older. Relevant outcomes were incidence of herpes zoster (primary outcome), herpes zoster ophthalmicus, post-herpetic neuralgia, quality of life, adverse events, and death.

**Results:**

27 studies (22 randomised controlled trials) including 2 044 504 patients, along with 18 companion reports, were included after screening 2037 titles and abstracts, followed by 175 full text articles. Network meta-analysis of five randomised controlled trials found no statistically significant differences between the live attenuated vaccine and placebo for incidence of laboratory confirmed herpes zoster. The adjuvant recombinant subunit vaccine, however, was statistically superior to both the live attenuated vaccine (vaccine efficacy 85%, 95% credible interval 31% to 98%) and placebo (94%, 79% to 98%). Network meta-analysis of 11 randomised controlled trials showed the adjuvant recombinant subunit vaccine to be associated with statistically more adverse events at injection sites than the live attenuated vaccine (relative risk 1.79, 95% credible interval 1.05 to 2.34; risk difference 30%, 95% credible interval 2% to 51%) and placebo (5.63, 3.57 to 7.29 and 53%, 30% to 73%, respectively). Network meta-analysis of nine randomised controlled trials showed the adjuvant recombinant subunit vaccine to be associated with statistically more systemic adverse events than placebo (2.28, 1.45 to 3.65 and 20%, 6% to 40%, respectively).

**Conclusions:**

Using the adjuvant recombinant subunit vaccine might prevent more cases of herpes zoster than using the live attenuated vaccine, but the adjuvant recombinant subunit vaccine also carries a greater risk of adverse events at injection sites.

**Protocol registration:**

Prospero CRD42017056389.

## Introduction

Herpes zoster, or shingles, is a neurocutaneous disease that occurs through reactivation of latent varicella zoster virus (the virus that causes chicken pox).^1^ A quarter of the population is at risk of developing herpes zoster during their lifetime,^2^ ^3^ ^4^ ^5^ ^6^ and two thirds of people with the disease are aged 50 years or older.^7^ The morbidity from herpes zoster increases with age.^8^ A systematic review reported higher case fatality rates for those aged 65 or more (61 per 100 000) compared with those aged 45-65 (2 per 100 000).^1^ In most high income countries, a live attenuated, injectable vaccine is available for the prevention of herpes zoster in adults aged 50 and older.^9^ However, vaccine efficacy decreases in those aged 70 and more,^10^ ^11^ and vaccine use is contraindicated in those with immunosuppression.^12^ ^13^ Recently, a new adjuvant recombinant subunit vaccine against herpes zoster has been approved in Canada,^14^ ^15^ the United States,^16^ Europe, and Japan.^17^

A Cochrane review compared the live attenuated vaccine with the new adjuvant recombinant subunit vaccine but excluded trials with participants aged less than 60 years as well trials with immunosuppressed people.^18^ As a result, one of the largest randomised controlled trials with 22 000 participants aged 50-59 years was excluded.^11^ The review also excluded observational studies, which are important for the examination of vaccine safety.^19^ Furthermore, since no head-to-head trials have compared the live attenuated vaccine with the new adjuvant recombinant subunit vaccine, an analysis that indirectly compares the herpes zoster vaccines through the common comparator placebo is required.

In this systematic review and network meta-analysis we compared the efficacy, effectiveness, and safety of the live attenuated herpes zoster vaccine with the adjuvant recombinant subunit vaccine, placebo, or no vaccine in those aged 50 years and older.

## Methods

### Protocol

A protocol was prepared in accordance with the Cochrane Handbook^20^ and the Preferred Reporting Items for Systematic Reviews and Meta-analysis for Protocols (PRISMA-P).^21^ All members of the team, as well as the review commissioners from the National Advisory Committee on Immunizations (NACI) and the Public Health Agency of Canada reviewed the draft protocol. The final version of the protocol was registered with PROSPERO.^22^ Results are reported using the PRISMA extension to network meta-analysis^23^ and the International Society for Pharmacoeconomics and Outcomes Research tool.^24^ ^25^ ^26^ ^27^ ^28^

### Eligibility criteria

We defined study eligibility criteria using the PICOS (population, intervention, comparator, outcome, and study) design approach^29^:

*Population*—adults aged 50 years and older

*Intervention*—live attenuated injectable herpes zoster vaccine

*Comparator(s)*—adjuvant recombinant subunit herpes zoster vaccine, placebo, or no vaccine. Studies that compared the same vaccine at different dosages, potencies, and routes of being administered were eligible for inclusion. We excluded experimental herpes zoster vaccines

*Outcomes*—vaccine efficacy for prevention of herpes zoster (primary outcome) was assessed on the basis of estimates from randomised controlled trials. Secondary outcomes included vaccine efficacy and effectiveness against post-herpetic neuralgia and herpes zoster ophthalmicus; quality of life measured using the EuroQol (EQ5D), Health Utilities Index Mark 2 (HUI2), Health Utilities Index Mark 3 (HUI3), or Short Form 6 Dimensions (SF6D); and vaccine safety, including adverse events at the injection site, systemic adverse events, serious adverse events, withdrawal of participants as a result of adverse events, potential immune mediated diseases, and death. Supplementary appendix S1 defines the study outcomes.

*Study designs—*experimental trials (randomised controlled, quasi-randomised controlled, non-randomised controlled), quasi-experimental studies (interrupted time series, controlled before and after), and observational studies (cohort, case-control) were eligible for inclusion. We excluded study designs without a comparator group (eg, case series, cross sectional), reviews, and pooled analyses.

All study periods and durations of follow-up were eligible. No other limitations, including language and location of publication, were imposed; both published and unpublished literature were eligible for inclusion.

### Information sources and literature search

One librarian (Elise Cogo) developed comprehensive literature searches of electronic bibliographic databases for Medline, Embase, and the Cochrane Library. Another librarian (Jessie McGowan) then reviewed the search strategy using the Peer Review of Electronic Search Strategies checklist.^30^ (Appendix S2 provides the combined search strategy for all three databases.) A library technician (Alissa Epworth) searched the electronic databases from inception to 19 January 2017, exported citations into an EndNote library, and removed duplicates. AE also conducted a supplementary search of grey literature (ie, studies that are difficult to locate and unpublished studies) using the guide produced by the Canadian Agency for Drugs and Technologies in Health.^31^ Grey literature sources that were searched included study registries (eg, ClinicalTrials.gov), grey literature databases (eg, SIGLE), conference abstracts, and theses and dissertations (see appendix S3). The reference lists of included studies and relevant reviews were scanned^9^ ^18^ and experts from NACI consulted on specific content.

### Screening process

To ensure reliability, the research team screened a random sample of 50 titles and abstracts before actual screening.^20^ As the inter-rate agreement was 76%, the reviewers independently screened titles and abstracts in pairs (WZ, RC, PK, VN, MG, RW, JPS). The inter-rater agreement for title and abstract screening was 91%. A random sample of 100 full text articles were initially screened to establish at least 75% inter-rater agreement before screening of full texts. Pairs of reviewers (WZ, RC, PK, VN, MG, RW, JPS) then independently screened full text articles; the inter-rater agreement was 80%. A third reviewer resolved discrepancies, with input from experts on clinical content (SES, AVP) as needed. An online screening tool, Synthesi.SR, developed by the Knowledge Translation Program, was used for all levels of screening and training exercises.^32^

### Data items and abstraction process

We extracted data on study characteristics (eg, duration of follow-up, study design, country, multicentre sites versus single site), patient characteristics (eg, mean age, age range, history of herpes zoster, history of chickenpox related comorbidities, overall health status according to the NACI criteria for immune compromising conditions (see appendix S4)), and outcome measures (herpes zoster incidence, post-herpetic neuralgia defined as pain continuing for 90 days or longer after the onset of a rash, herpes zoster ophthalmicus, adverse events, quality of life, and death). Outcome results were only abstracted for the longest duration of follow-up, as this is the most conservative approach.^20^ A draft data collection form was established after consultation with the research team, including clinicians and methodologists.

Before data abstraction, the review team tried out the data abstraction form on a random sample of five articles.^20^ Subsequently, pairs of reviewers (WZ, RC, PK, VN, MG, RW, JPS) independently abstracted the included studies. A third reviewer (WZ) compiled the statistical files and resolved discrepancies between reviewers, ensuring data accuracy and consistency. Authors were contacted for missing information and clarification (eg, whether patient populations overlapped in publications that used the same administrative databases).

### Risk of bias assessment

Pairs of reviewers (PK, VN, MG) independently appraised risk of bias in each study using the Cochrane risk of bias tool^33^ for randomised controlled trials and quasi-randomised controlled trials, the Newcastle Ottawa Scale^34^ for observational studies, and the Cochrane Effective Practice and Organisation of Care Risk-of-Bias Tool^35^ for non-randomised trials and quasi-experimental studies. Only reviewers experienced in using the tools were involved at this step. A third reviewer (RC) consistently resolved discrepancies.

### Statistical analysis

A bayesian approach was used to conduct meta-analyses and network meta-analyses.^36^ Analyses were carried out in a bayesian environment, which allows for the inclusion of an informative prior in the model variable for the between study variance^37^; as well as the implementation of hierarchical models that preserve the treatment-dose relation in a dose effects analysis.^38^ Pairwise meta-analysis was conducted when at least two studies examined the same intervention and comparator for a particular outcome. When the treatment nodes formed a connected network of evidence, we did a network meta-analysis to compare the different herpes zoster vaccines using the common comparator, placebo. The treatment nodes included in the analysis were selected by methodologists (ACT, AAV) and clinical experts (SES, AVP), who categorised vaccine dosages across the studies and which were based on the recommended dosage according to Health Canada and vaccine product monographs (table 1).^39^ ^40^ To ensure the transitivity assumption was upheld, we plotted the central tendencies (eg, means, medians) of study and patient characteristics for each treatment comparison for visual inspection.^41^ It was not possible to examine the consistency assumption, since no closed loops were included in the networks.^42^ ^43^ ^44^ ^45^

**Table 1:** Recommended dosage for herpes zoster vaccines

Vaccine	Vaccine type	Recommended dosage	Dosage categories	Source
Zostavax, Merck Canada, Canada	Live attenuated	Single dose comprises entire contents of vial (about 0.65 mL, containing varicella zoster virus ≥19 400 plaque forming units (PFU)). Administered by subcutaneous injection	Low dose: 3500-10 000 PFU×1 dose. Standard dose: 19 400-23 000 PFU×1 dose. High dose: 40 000-200 000 PFU×1 dose	Product monograph^39^
Shingrix, GlaxoSmithKline, Canada	Adjuvant recombinant subunit	Two doses 0.5 mL each (contains 50 μg varicella zoster virus glycoprotein E) with initial dose at month 0 followed by second dose anytime between 2 and 6 months later. Second dose is important to ensure maximum vaccine efficacy and duration of protection against herpes zoster. Administered by intramuscular injection, preferably in deltoid muscle	Low dose: 25 μg×2 doses. Standard dose: 50 μg×2 doses. High dose: 100 μg×2 doses	Product monograph^40^

To account for anticipated methodological and clinical heterogeneity across studies and to achieve the highest generalisability in the pooled treatment effects, we applied a random effects model in both meta-analysis and network meta-analysis. Furthermore, to increase power in the heterogeneity estimation for each network, we assumed a common within network τ^2^ across treatment comparisons in both meta-analysis and network meta-analysis models. This assumption was clinically reasonable as similar treatments were included.

Analyses were conducted using OpenBUGS (version 3.2.3 rev 1012) assuming a half-normal prior for the between study standard deviation (τ∼*N*(0,1),τ>0) for the primary analysis, and using vague priors for all remaining model variables. The primary outcome of herpes zoser incidence was also analysed with an informative prior as a sensitivity analysis.^37^ ^46^ We excluded from the analysis those studies reporting zero events across all treatment arms for a particular outcome (see appendix S5). The Markov Chain Monte Carlo method was used to calculate the median odds ratios and 95% credible intervals. For the comparison of the adjuvant recombinant subunit vaccine with live attenuated vaccine we used the mean control event rate for placebo and the treatment event rate for herpes zoster to transform odds ratio to relative risks. We used established formulas to convert the results for efficacy and effectiveness to vaccine efficacy and effectiveness.^47^ Vaccine efficacy and effectiveness was calculated for doctor or laboratory confirmed herpes zoster, suspected herpes zoster, herpes zoster ophthalmicus, and post-herpetic neuralgia.

We calculated the 95% prediction interval for all network meta-analysis estimates; this statistic predicts the interval within which the results of a future study might lie.^48^ To examine the hierarchy of vaccines, we used the Surface Under the Cumulative RAnking curves, along with the 95% credible intervals^49^ and plotted these using the rank heat plot.^50^ To examine potential publication bias and small study effects, we produced an adjusted funnel plot for outcomes including at least 10 studies.^51^ To examine the dose effects of the vaccine, we applied a hierarchical model—the exchangeable subnodes model with subnode consistency.^38^

To examine the robustness of results, we planned several additional analyses a priori: sensitivity analysis restricted to studies with a low risk of bias, subgroup analysis of age (eg, 50-64 years *v* ≥65 years), health status (healthy adults with no immune compromising conditions referred to as immunocompetent versus adults with at least one immune comprising condition referred to as immunocompromised), sex (women versus men), herpes zoster status (previous herpes zoster infection versus herpes zoster naïve), herpes zoster ophthalmicus (previous herpes zoster ophthalmicus versus none), vaccine status (previous vaccination with herpes zoster live attenuated vaccine versus vaccine naïve), and study design (randomised controlled trials versus non-randomised studies versus randomised controlled trials+non-randomised studies). When data on 10 or more studies were available we examined the duration of follow-up and age distribution through network meta-regression analysis, which was only possible for two outcomes: adverse events at injection sites and suspected herpes zoster. We only considered additional analyses when the number of studies was greater than the number of treatments for each outcome.

### GRADE appraisal

Technical leads and clinical expert members of the NACI Working Group graded the quality (or certainty) of the available evidence. The results of the GRADE assessments are in the report released by NACI.^9^

### Patient and public involvement

No patients or the public were involved in setting the research question or outcome measures, nor were they involved in the design and implementation of the study. This is because the commissioning agency and the primary knowledge user, NACI, did not allow for patient engagement, as they had an expedited timeline to make policy decisions on herpes zoster vaccines.

## Results

### Literature search

**Fig 1:**
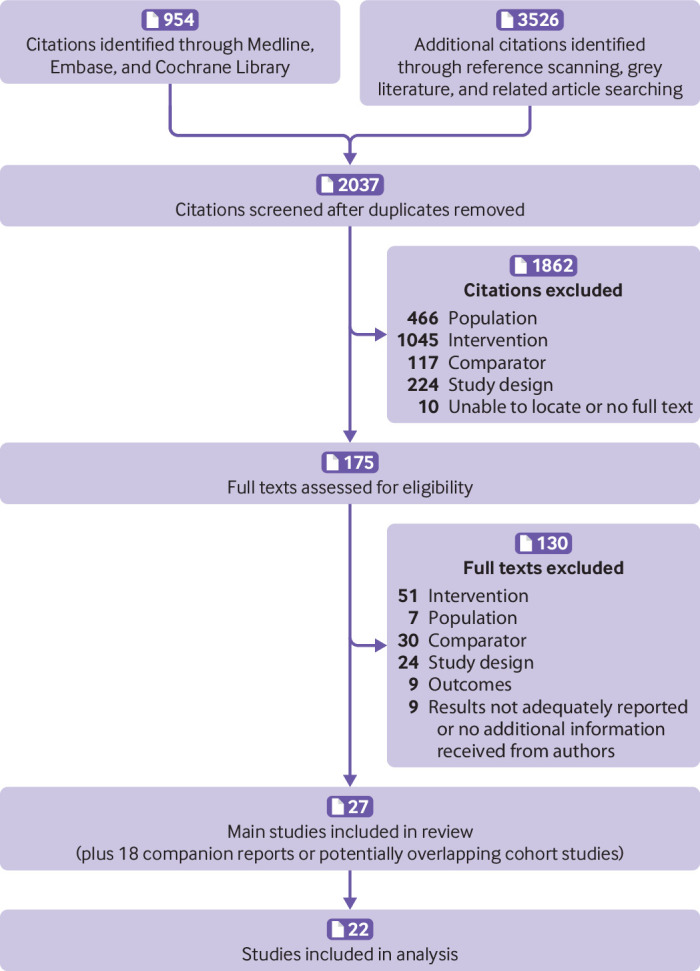
Study flow diagram

After screening 2037 titles and abstracts and 175 full text articles, 27 studies^10^ ^11^ ^52^ ^53^ ^54^ ^55^ ^56^ ^57^ ^58^ ^59^ ^60^ ^61^ ^62^ ^63^ ^64^ ^65^ ^66^ ^67^ ^68^ ^69^ ^70^ ^71^ ^72^ ^73^ ^74^ ^75^ ^76^ providing data on 2 044 504 patients, and 18 companion reports,^77^ ^78^ ^79^ ^80^ ^81^ ^82^ ^83^ ^84^ ^85^ ^86^ ^87^ ^88^ ^89^ ^90^ ^91^ ^92^ ^93^ ^94^ met the eligibility criteria (fig 1). Twenty two studies^10^ ^11^ ^52^ ^53^ ^54^ ^55^ ^56^ ^60^ ^61^ ^62^ ^63^ ^64^ ^65^ ^66^ ^67^ ^68^ ^70^ ^71^ ^72^ ^73^ ^76^ ^95^ were included in the statistical analysis. The remaining five studies^57^ ^58^ ^59^ ^74^ ^75^ compared the same vaccine by routes of vaccination, handling of the vaccines, storage of the vaccines, or by time between doses (see summary in appendix S6). Of the included studies, two were unpublished trial registry data.^68^ ^77^ All were reported in English. Twelve authors were contacted for additional clarification or missing data and nine responded with data clarifications but no additional data.

### Study and patient characteristics

The 27 studies were published between 1998 and 2017: nine in North America (33%), five in Europe (19%), two in Asia (7%), and 11 across several geographical regions (41%) (table 2, also see appendix S7). Twenty three of the trials were conducted across multiple study centres (74%) and 22 studies used a randomised controlled trial study design (81%). The average study duration was 30.9 months (SD 22.3 months). The live attenuated vaccine was the most commonly examined vaccine type (70%) and the incidence of suspected or confirmed herpes zoster was the most commonly examined outcome (81%).

**Table 2:** Characteristics of the 27 included studies

Characteristics	No (%) of studies
Publication year:	
1998-2002	1 (4)
2003-07	3 (11)
2008-12	7 (26)
2013-17	16 (59)
Geographical region:	
Asia	2 (7)
Europe	5 (19)
North America	9 (33)
Multi-continent	11 (41)
Study setting:	
Single centre	3 (11)
Multicentre	20 (74)
Not reported	4 (15)
Study design:	
Case-control	1 (4)
Non-randomised controlled trial	1 (4)
Cohort	3 (11)
Randomised controlled trial	22 (81)
Study duration (months)*†:	
0-12	5 (19)
13-24	7 (26)
25-36	6 (22)
37-48	4 (15)
49-60	2 (7)
>60	2 (7)
Not reported	1 (4)
Frequency of interventions examined:	
Live attenuated herpes zoster vaccine	19 (70)
Adjuvant recombinant subunit herpes zoster vaccine	7 (26)
Varicella zoster vaccine	1 (4)
Live attenuated herpes zoster vaccine and pneumovax 23 vaccine	1 (4)
Outcomes†‡:	
Herpes zoster suspected/confirmed	22 (81)
Herpes zoster ophthalmicus	3 (11)
Post-herpetic neuralgia	4 (15)
Injection site adverse event	21 (78)
Systemic adverse event	17 (63)
Serious adverse event	20 (74)
Withdrawals related to adverse event	17 (63)
Potential immune mediated disease	4 (15)
Death	20 (74)
Quality of life	0 (0)

* Mean 30.9 (SD 22.3) months.

† Also, see Appendix S1 for definitions.

‡ Not all studies could be included in pooled analysis.

Twenty three studies (85%) included patients who were immunocompetent, and 22 studies (81%) included a sample consisting of more than 50% women (table 3, also see appendix S8). Twenty four studies (89%) included patients with no history of herpes zoster, and 18 studies included participants with documented varicella infection (67%).

**Table 3:** Characteristics of participants in the 27 included studies

Characteristics	No (%) of studies
Age group (years):	
≥50	11 (41)
≥60	10 (37)
≥70	2 (7)
50-59	1 (4)
50-70	1 (4)
60-70	1 (4)
60-88	1 (4)
Immune related health:	
Immunocompetent†	23 (85)
Immunodeficient	2 (7)
Mixed	2 (7)
Proportion women (%):	
<50	5 (19)
50-60	13 (48)
61-75	9 (33)
History of herpes zoster:	
Yes	1 (4)
No	24 (89)
Not reported	2 (7)
History of varicella zoster:	
Yes	18 (67)
No	1 (4)
Not reported	8 (30)

### Risk of bias results

**Fig 2:**
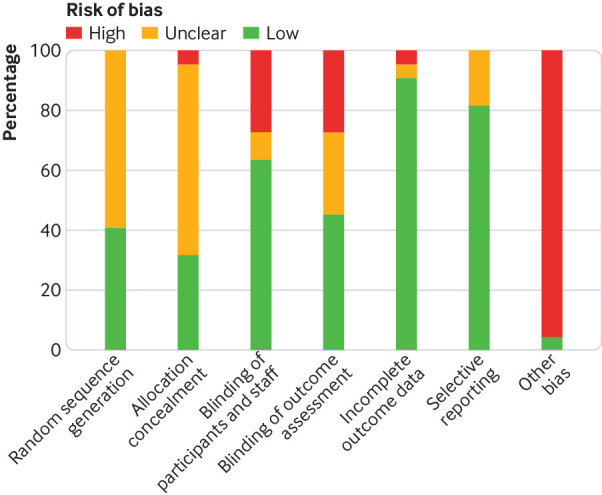
Distribution of Cochrane Collaboration Risk of Bias assessment (n=22 randomised controlled trials). The “other bias” item was scored as high risk of bias when studies were funded by private industries and included authors who are employed by vaccine manufacturers

Thirteen of the 22 included randomised controlled trials had unclear risk of bias from inadequate reporting of random sequence generation (59%) and 14 from inadequate reporting of allocation concealment (64%). In addition, 21 studies had a high risk of “other” biases (95%), since most of the studies were funded by private industry, and the coauthors included employees from vaccine manufacturers (fig 2, also see appendix S9). The three cohort studies and one case-control study had high to moderate risk of bias for representativeness of the exposed cohort, and for comparability of cohorts in three of the four studies (see appendix S10). The non-randomised trial had an unclear risk of bias for random sequence generation, allocation concealment, blinding, and contamination, as well as a high risk of “other” bias (see appendix S11).

### Statistical analysis results

**Fig 3:**
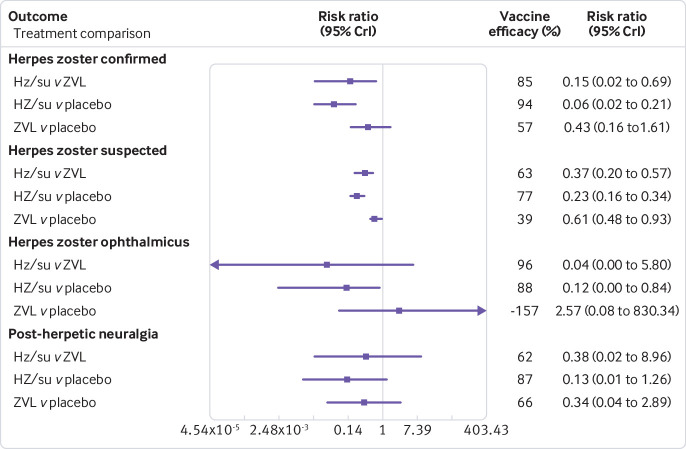
Forest plot of estimated results from meta-analysis and network meta-analysis of vaccine efficacy outcomes in reducing cases of herpes zoster, herpes zoster ophthalmicus, and post-herpetic neuralgia. HZ/su=herpes zoster adjuvant recombinant subunit vaccine; ZVL=herpes zoster live attenuated vaccine

**Fig 4:**
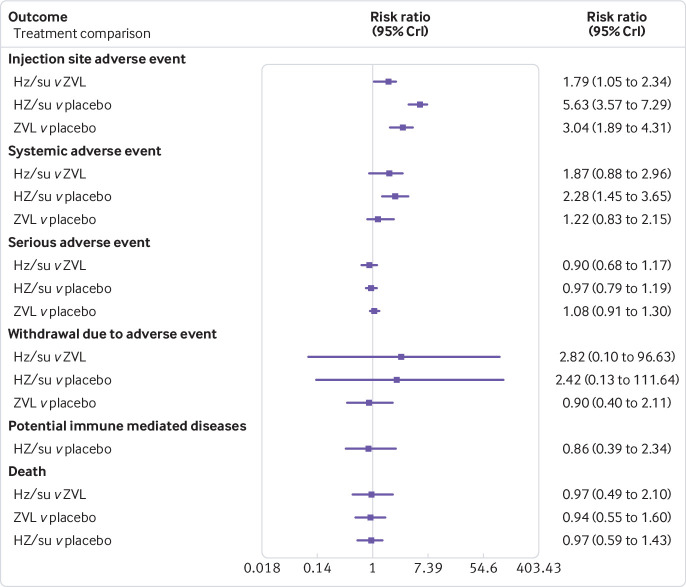
Forest plot of estimated results from meta-analysis and network meta-analysis of safety outcomes including, injection site, systemic, and serious adverse events, and withdrawal due to adverse events, as well as potential immune mediated diseases and death. HZ/su=herpes zoster adjuvant recombinant subunit vaccine; ZVL=herpes zoster live attenuated vaccine

Across all analyses, only three comparators were included: live attenuated vaccine, adjuvant recombinant subunit vaccine, and placebo/no vaccine. The transitivity plots suggested that the effect modifiers were balanced across the treatments when these were reported, however, several of the analyses did not have information on all comparisons in the network (see appendix S12). Comparison adjusted funnel plots were generated for the cases of suspected herpes zoster and injection site adverse events outcomes (see appendix S13). Only 11 unique trials existed for both of these outcomes, resulting in low power to examine publication bias and small study effects. Although the funnel plot for injection site adverse events was considered to be symmetrical, the funnel plot for suspected herpes zoster was asymmetrical. Further examination revealed three trials^61^ ^66^ ^70^ with large standard errors and negative centred effect sizes, so a sensitivity analysis was done excluding these three trials. The results in table 4 and table 5 and in figure 3 and figure 4 are based on randomised controlled trials that included immunocompetent or immunocompromised patients, or both.

**Table 4:** Summary of main results using randomised controlled trials only and intention to treat sample: meta-analyses and network meta-analyses

Treatment comparison, reference	No of studies (No of patients)	Study group (No of events/total No)		Odds ratio from direct and indirect comparisons (95% CrI) (95% PrI indirect comparison only)	Risk ratio		Vaccine efficacy % (95% CrI)
		Treatment	Control		Direct comparison (meta-analysis) (95% CrI)	Indirect comparison (95% CrI) (95% PrI)	
**Doctor or laboratory confirmed herpes zoster cases:** 5 RCTs, 90 605 participants, average follow-up 28 (range 2-44) months							
HZ/su *v* ZVL	—	—	—	0.15 (0.02 to 0.68) (0.01 to 1.42)*	—	0.15 (0.02 to 0.69) (0.01 to 1.41)*	85 (31 to 98)*
HZ/su *v* placebo^56^ ^62^ ^78^	2 (29 311)	32/14 648	458/14 663	0.06 (0.02 to 0.21)*	0.06 (0.02 to 0.21)*	—	94 (79 to 98)*
ZVL *v* placebo^10^ ^11^ ^70^ ^80^-^87^	3 (61 294)	346/30 688	741/30 606	0.43 (0.15 to 1.63)	0.43 (0.16 to 1.61)	—	57 (−61 to 84)
Common within network between study variance	—	—	—	—	0.37 (0.02 to 3.02)	0.37 (0.02 to 3.10)	—
**Suspected herpes zoster cases:** 7 RCTs, 91 840 participants, average follow-up 20 (range 1-44) months							
HZ/su *v* ZVL	—	—	—	0.37 (0.20 to 0.57) (0.16 to 0.71)*	—	0.37 (0.20 to 0.57) (0.16 to 0.71)*	63 (43 to 80)*
HZ/su *v* placebo^56^ ^62^ ^78^	2 (29 311)	150/14 648	643/14 663	0.23 (0.15 to 0.33)*	0.23 (0.16 to 0.34)*	—	77 (66 to 84)*
ZVL *v* placebo^10^ ^11^ ^61^ ^66^ ^70^ ^80^-^87^	5 (62 529)	597/31 307	1000/31 222	0.60 (0.47 to 0.93)*	0.61 (0.48 to 0.93)*	—	39 (7 to 52)*
Common within network between study variance	—	—	—	—	0.01 (0.00 to 0.46)	0.01(0.00 to 0.49)	—
**Herpes zoster ophthalmicus cases:** 2 RCTs, 14 209 participants, average follow-up 25 (range 2-60) months							
HZ/su *v* placebo†^56^ ^78^	1 (13 900)	1/6950	6/6950	0.12 (0.00 to 0.84)*	0.12 (0.00 to 0.84)*	-	88 (16 to 100)*
ZVL *v* placebo†^70^	1 (309)	1/207	0/102	2.57 (0.08 to 1293.66)	2.57 (0.08 to 830.34)	—	−157 (−129266 to 92)
Common within network between study variance	—	—	—	—	0.48 (0.00 to 5.07)	—	—
**Post-herpetic neuralgia:** 2 RCTs, 52 446 participants, average follow-up 26 (range 6-46) months							
HZ/su *v* placebo†^56^ ^78^	1 (13 900)	4/6950	28/6950	0.13 (0.04 to 0.35)*	0.13 (0.04 to 0.35)*	—	87 (65 to 96)*
ZVL *v* placebo†^10^ ^80^-^86^	1 (38 546)	27/19 270	80/19 276	0.33 (0.21 to 0.51)*	0.34 (0.21 to 0.51)*	—	66 (49 to 79)*
Common within network between study variance	—	—	—	—	0.46 (0.00 to 4.93)	—	—

CrI=credible interval; PrI=prediction interval; RCT=randomised controlled trial; HZ/su=herpes zoster adjuvant recombinant subunit vaccine; ZVL=herpes zoster live attenuated vaccine.

* P<0.05.

† Only one study included in comparison.

**Table 5:** Summary of findings for secondary safety outcomes

Treatment comparison, reference	No of studies (No of patients)	Study group (No of events/total No)		Odds ratio from direct and indirect comparisons (95% CrI) (95% PrI indirect comparison only)	Risk ratio		Vaccine efficacy % (95% CrI)
		Treatment	Control		Direct comparison (meta-analysis) (95% CrI)	Indirect comparison (95% CrI) (95% PrI)	
**Injection site adverse events**: 11 RCTs, 92 431 participants, average follow-up 27 (range 5-42) days							
HZ/su *v* ZVL	—	—	—	3.42 (1.09 to 12.50) (0.46 to 29.40)*	—	1.79 (1.05 to 2.34) (0.57 to 2.51)*	—
HZ/su *v* placebo^53^ ^55^ ^61^ ^77^	3 (29 499)	4064/14 798	575/14 701	14.28 (5.39 to 41.31)*	5.63 (3.57 to 7.29)*	—	—
ZVL *v* placebo^10^ ^11^ ^59^ ^60^ ^65^ ^67^ ^69^ ^72^	8 (62 932)	9081/31 478	2302/31 454	4.14 (2.14 to 7.60)*	3.04 (1.89 to 4.31)*	—	—
Common within network between study variance	—	—	—	—	0.57 (0.19 to 1.92)	0.57 (0.19 to 1.91)	—
**Systemic adverse events**: 9 RCTs, 91 196 participants, average follow-up 29 (range 7-42) days							
HZ/su *v* ZVL	—	—	—	2.37 (0.85 to 5.74) (0.45 to 10.13)	—	1.87 (0.88 to 2.96) (0.50 to 3.61)	—
HZ/su *v* placebo^53^ ^55^ ^61^ ^77^	3 (29 499)	3251/14 798	1427/14 701	2.97 (1.57 to 6.96)*	2.28 (1.45 to 3.65)*	—	—
ZVL *v* placebo^10^ ^11^ ^59^ ^67^ ^69^ ^72^ ^79^-^86^	6 (61 697)	4837/30 859	4530/30 838	1.27 (0.81 to 2.70)	1.22 (0.83 to 2.15)	—	—
Common within network between study variance	—	—	—	—	0.18 (0.00 to 1.52)	0.19 (0.00 to 1.56)	—
**Serious adverse events**: 8 RCTs, 103 899 participants, average follow-up 9 (range 1-49) months							
HZ/su *v* ZVL	—	—	—	0.89 (0.67 to 1.17) (0.60 to 1.29)	—	0.90 (0.68 to 1.17) (0.61 to 1.28)	—
HZ/su *v* placebo^55^ ^61^ ^77^	2 (29 311)	1842/14 648	1900/14 663	0.97 (0.79 to 1.20)	0.97 (0.79 to 1.19)	—	—
ZVL *v* placebo^10^ ^11^ ^60^ ^65^ ^69^ ^79^-^86^ ^94^	6 (74 588)	590/37 307	546/37 281	1.08 (0.91 to 1.32)	1.08 (0.91 to 1.30)	—	—
Common within network between study variance	—	—	—	—	0.00 (0.00 to 0.13)	0.00 (0.00 to 0.13)	—
**Withdrawal due to adverse events**: 6 RCTs, 35 678 participants, average follow-up 20 (range 8-34) months							
HZ/su *v* ZVL vaccine	—	—	—	2.87 (0.09 to 1161.44) (0.07 to 1365.37)	—	2.82 (0.10 to 96.63) (0.07 to 97.83)	—
HZ/su *v* placebo†^53^	1 (188)	2/150	0/38	2.45 (0.13 to 1115.45)	2.42 (0.13 to 111.64)	—	—
ZVL *v* placebo^11^ ^65^ ^69^ ^72^ ^86^ ^94^	5 (35 490)	45/17 760	50/17 730	0.90 (0.40 to 2.12)	0.90 (0.40 to 2.11)	—	—
Common within network between study variance	—	—	—	—	0.20 (0.00 to 2.33)	0.19 (0.00 to 2.39)	—
**Potential immune mediated diseases**: 2 RCTs, 29 311 participants, average follow-up 47 (range 45-48) months							
HZ/su *v* placebo^55^ ^61^ ^77^	2 (29 311)	170/14 648	194/14 663	0.86 (0.38 to 2.38)	0.86 (0.39 to 2.34)	—	—
Common within network between study variance	—	—	—	—	0.06 (0.00 to 2.94)	—	—
**Death**: 7 RCTs, 102 718 participants, average follow-up 26 (range 2-48) months							
HZ/su *v* ZVL	—	—	—	0.97 (0.49 to 2.15) (0.35 to 2.84)	—	0.97 (0.49 to 2.10) (0.35 to 2.75)	—
HZ/su *v* placebo^55^ ^61^ ^77^	2 (29 311)	593/14 648	633/14 663	0.94 (0.54 to 1.62)	0.94 (0.55 to 1.60)	—	—
ZVL *v* placebo^10^ ^11^ ^59^ ^69^ ^79^-^86^ ^94^	5 (73 407)	837/36 715	844/36 692	0.97 (0.58 to 1.45)	0.97 (0.59 to 1.43)	—	—
Common within network between study variance	—	—	—	—	0.03 (0.00 to 0.88)	0.03 (0.00 to 0.93)	

CrI=credible interval; PrI=prediction interval; RCT=randomised controlled trial; HZ/su=herpes zoster adjuvant recombinant subunit vaccine; ZVL=herpes zoster live attenuated vaccine.

* P<0.05.

† Only one study included in comparison.

### Efficacy and effectiveness results

#### Laboratory or doctor confirmed herpes zoster

Five randomised controlled trials including 90 605 immunocompetent and immunocompromised patients were included in the network meta-analysis of laboratory or doctor confirmed cases of herpes zoster. The adjuvant recombinant subunit vaccine was statistically superior to placebo (vaccine efficacy 94%, 95% credible interval 79% to 98%) and the live attenuated vaccine (85%, 31% to 98%), whereas the live attenuated vaccine was not statistically different from placebo (table 4 and table 5). These results were similar in the sensitivity analysis restricted to immunocompetent patients only (see appendix S14). Four randomised controlled trials were included in the dose effects analysis, and only the adjuvant recombinant subunit vaccine was statistically superior to placebo (see appendix S23), which was consistent in additional network meta-analysis restricted to four randomised controlled trials including patients with no history of herpes zoster, and restricted to four randomised controlled trials with low risk of bias on random sequence generation. Additional analyses based on all study designs (five randomised controlled trials and one cohort study) and the efficacy comparison using informative priors^37^ (five randomised controlled trials) found that the adjuvant recombinant subunit vaccine was statistically superior to placebo and the live attenuated vaccine, and that the live attenuated vaccine was statistically superior to placebo.

#### Suspected herpes zoster

Seven randomised controlled trials including 91 840 immunocompetent and immunocompromised patients were included in the network meta-analysis of suspected herpes zoster infection. The adjuvant recombinant subunit vaccine was statistically superior to placebo (vaccine efficacy 77%, 95% credible interval 66% to 84%) and the live attenuated vaccine (63%, 43% to 80%), and the live attenuated vaccine was statistically superior to placebo (39%, 7% to 52%; table 4 and table 5). These results were consistent in additional analyses, including all study designs (seven randomised controlled trials, three cohort studies, one case-control study), network meta-regression on duration of follow-up (seven randomised controlled trials, three cohort studies, one case-control study), using an informative prior^37^ (seven randomised controlled trials), sensitivity analysis restricted to immunocompetent (and potentially immunocompetent) patients (six randomised controlled trials), sensitivity analysis restricted to immunocompetent patients (four randomised controlled trials), as well as a sensitivity analysis excluding three trials^61^ ^66^ ^70^ to explain the asymmetrical funnel plot. (Also see appendix S15.) However, in sensitivity analysis restricted to four randomised controlled trials with a low risk bias on random sequence generation, the live attenuated vaccine was no longer statistically different from placebo. In the dose analysis, only standard doses of the live attenuated vaccine and adjuvant recombinant subunit vaccine were statistically significant compared with placebo (see appendix S23).

#### Herpes zoster ophthalmicus

Only two randomised controlled trials including 14 209 immunocompetent and immunocompromised patients reported on cases of herpes zoster ophthalmicus; therefore no network meta-analysis was possible. The pairwise meta-analysis showed that the adjuvant recombinant subunit vaccine was statistically superior to placebo (13 900 patients, vaccine efficacy 88%, 95% credible interval 16% to 100%) and no statistically significant difference was observed between the live attenuated vaccine and placebo (309 patients) (table 4 and table 5, also see appendix S16).

#### Post-herpetic neuralgia

Only two randomised controlled trials including 52 446 immunocompetent patients reported on post-herpetic neuralgia, and the meta-analysis found a statistically significant difference between the adjuvant recombinant subunit vaccine and placebo (13 900 patients, vaccine efficacy 87% 95% credible interval 65% to 96%), as well as between the live attenuated vaccine and placebo (38 546 patients, 66%, 49% to 79%) (table 4 and table 5, also see appendix S17).

### Adverse events

*Injection sites—*11 randomised controlled trials including 92 431 immunocompetent and immunocompromised patients were included in the main network meta-analysis of adverse events at injection sites (eg, redness or swelling). The adjuvant recombinant subunit vaccine was statistically inferior to the live attenuated vaccine (relative risk 1.79, 95% credible interval 1.05 to 2.34) and placebo (5.63, 3.57 to 7.29), and the live attenuated vaccine was statistically inferior to placebo (3.04, 1.89 to 4.31; table 4 and table 5). The adjuvant recombinant subunit vaccine was not, however, statistically different from the live attenuated vaccine in additional analyses including patients with no history of herpes zoster (10 randomised controlled trials), network meta-regression on duration of follow-up (11 randomised controlled trials), sensitivity analysis restricted to randomised controlled trials with a low risk of bias on random sequence generation (six randomised controlled trials), sensitivity analysis restricted to immunocompetent and potentially immunocompetent patients (10 randomised controlled trials), and sensitivity analysis restricted to only immunocompetent patients (six randomised controlled trials) (see appendix S18). In sensitivity analysis restricted to five randomised controlled trials with a low risk of bias on allocation concealment, only the adjuvant recombinant subunit vaccine was statistically inferior to placebo. In the dose effect analysis, only the live attenuated vaccine was statistically inferior to placebo (see appendix S23).

*Systemic*—nine randomised controlled trials including 91 196 immunocompetent and immunocompromised patients were included in the network meta-analysis of systemic adverse events (eg, generalised reactions such as headache and myalgia). The adjuvant recombinant subunit vaccine was statistically inferior to placebo (relative risk 2.28, 95% credible interval 1.45 to 3.65; table 4 and table 5). This was consistent in the additional analysis restricted to eight randomised controlled trials including immunocompetent and potentially immunocompetent patients (see appendix S19). None of the comparisons were, however, statistically significant in the dose effect analysis (see appendix S23) or in any of the other additional analyses.

*Serious*—eight randomised controlled trials including 103 899 immunocompetent and immunocompromised patients were included in the network meta-analysis of serious adverse events (eg, requiring hospital admission), and none of the comparisons were statistically significant (table 4 and table 5). This was consistent in the dose effect analysis (see appendix S23) and in all additional analyses (see appendix S20).

*Withdrawal due to adverse events*—six randomised controlled trials including 35 678 immunocompetent and immunocompromised patients were included in the network meta-analysis of withdrawal due to adverse events, and none of the comparisons were statistically significant (table 4 and table 5). This was consistent in the dose effect analysis (see appendix S23) and in all additional analyses (see appendix S21).

### Potential immune mediated diseases

Only two randomised controlled trials including 29 311 immunocompetent patients were included in the meta-analysis on potential immune mediated diseases (table 4 and table 5); there was no statistical difference between the adjuvant recombinant subunit vaccine and placebo. No additional analyses were possible for this outcome.

### Death

Seven randomised controlled trials including 102 718 immunocompetent and immunocompromised patients were included in the network meta-analysis for observed death during the study period, and none of the comparisons were statistically significant (table 4 and table 5). This was consistent in the dose effect analysis (see appendix S23) and in all additional analyses (see appendix S22).

### Rank heat plot

**Fig 5:**
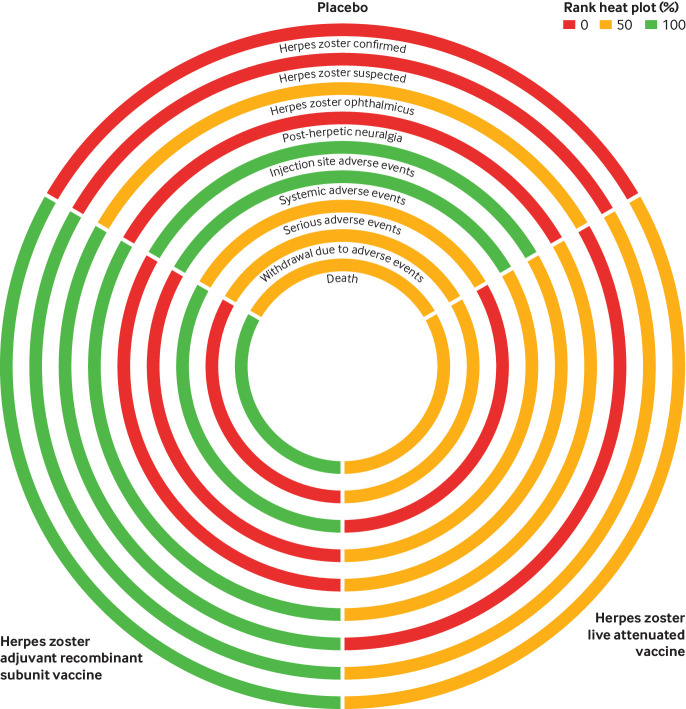
Rank heat plot, summarising treatment hierarchy across all outcomes. Each circle represents an outcome and has been sectioned into the three interventions, adjuvant, recombinant subunit herpes zoster vaccine, live attenuated herpes zoster vaccine, and placebo. The performance of a particular treatment for each of the outcomes is interpreted based on three colours (worst to best): red (0%), yellow (50%), and green (100%)

The rank heat plot suggests that the adjuvant recombinant subunit vaccine is the most effective vaccine against herpes zoster, herpes zoster ophthalmicus, and post-herpetic neuralgia, with fewer serious adverse events and deaths than using the live attenuated vaccine or placebo. The live attenuated vaccine is safer for injection site and systemic adverse events, with fewer withdrawals due to adverse events compared with the adjuvant recombinant subunit vaccine (fig 5).

## Discussion

The results suggest that the adjuvant recombinant subunit vaccine is superior to the live attenuated vaccine for the prevention of herpes zoster infection, as measured by laboratory or doctor confirmed cases and suspected cases. Owing to a lack of data, the superiority of the adjuvant recombinant subunit vaccine over the live attenuated vaccine in reducing the number of cases of herpes zoster ophthalmicus and post-herpetic neuralgia is inconclusive. The adjuvant recombinant subunit vaccine was, however, associated with more adverse events at injection sites than the live attenuated vaccine. On the basis of the limited indirect evidence available, no statistically significant differences were shown between the vaccines for serious adverse events, withdrawals due to adverse events, potential immune mediated diseases, and death. A current randomised controlled trial comparing the immunogenicity and safety of the adjuvant recombinant subunit vaccine with the live attenuated vaccine will provide direct evidence on the relative safety of the two vaccines.^96^ As such, the results of this systematic review will need to be updated with evidence from this trial, as well as any additional new trials completed after the literature search. Differences between the dosages of the vaccine were not observed, suggesting the standard doses are appropriate for clinical use.

### Strengths and limitations of this study

This systematic review has several strengths. As no trials currently compare the safety and effectiveness of the adjuvant recombinant subunit vaccine and live attenuated vaccine directly, this systematic review tackles an important evidence gap by indirectly comparing the two available vaccines using valid meta-analysis methods to inform clinicians and policy makers. We followed international guidelines on the conduct and reporting of systematic reviews and network meta-analyses, including the Cochrane Handbook,^20^ International Society for Pharmacoeconomics and Outcomes Research tool,^24^ ^25^ ^26^ and PRISMA statements.^21^ ^27^ ^28^ Several limitations do, however, relate to the included studies as they might have been affected by several types of bias, such as from inadequate reporting of random sequence generation, allocation concealment, and comparability of cohorts. Also, several of the planned analyses were not conducted owing to insufficient data, such as subgroup analysis on the age of participants, network meta-regression for duration of follow-up, or analyses restricted to immunocompromised patients across all outcomes. Furthermore, asymmetry was observed in the funnel plot for the suspected herpes zoster outcome, which is likely caused by variation in study characteristics; the sample sizes ranged from 309 to 762 patients in four studies and from 13 900 to 766 330 patients in another seven studies. After excluding three trials with large standard errors and negative centred effect sizes, the results from the meta-analysis and network meta-analysis were unchanged.

### Comparison with other studies

The findings reported here are consistent with a review of the evidence conducted by the Centers for Disease Control and Prevention, which recommended the adjuvant recombinant subunit vaccine for immunocompetent adults aged 50 years and older.^97^ Although a literature review of the evidence was carried out, the CDC did not conduct a systematic review or network meta-analysis. The CDC also did a cost analysis and found that the adjuvant recombinant subunit vaccine prevented more disease at lower overall costs than the live attenuated vaccine. However, an additional cost effectiveness analysis might provide further clarity. Importantly, the adjuvant recombinant subunit vaccine requires two doses to be administered intramuscularly at 0 and 2-6 months, whereas the live attenuated vaccine requires a single subcutaneous dose. As such, a comparison of cost, including vaccine route of being administered, as well as storage and handling is required. A retrospective cohort study published after the literature search found the live attenuated vaccine to be effective, but immunity waned after five years.^98^ The study also found that as people aged, the vaccine was less effective.

The results of this systematic review indicate that the adjuvant recombinant subunit vaccine is likely more effective than the live attenuated vaccine for preventing herpes zoster. Additionally, the dose effects analysis suggested that current standard doses are appropriate. As such, clinicians and policy makers can use these results to inform their recommendations about these vaccines to patients and the general public.

### Policy implications

Several unanswered questions remain. For example, it is unclear whether the adjuvant recombinant subunit vaccine is effective for immunocompromised people. It also remains to be confirmed whether the live attenuated vaccine is more effective than the adjuvant recombinant subunit vaccine for protection against post-herpetic neuralgia, whether a booster dose is required, whether the adjuvant recombinant subunit vaccine is comparatively cost effective considering it needs to be administered in two doses, and what the safety implications are for the recombinant subunit vaccine, given that it comprises an adjuvant. Furthermore, many of the studies had small sample sizes, especially the randomised controlled trials. Future trials should include larger sample sizes and examine quality of life, the incidence of herpes zoster ophthalmicus and post-herpetic neuralgia, and potential immune mediated diseases.

### Conclusions

The adjuvant recombinant subunit vaccine is likely superior to the live attenuated vaccine against herpes zoster; however, the adjuvant recombinant subunit vaccine also carries a greater risk of adverse events at injection sites. No statistically significant differences were identified between the two vaccines for serious adverse events, withdrawals related to adverse events, and death. Differences between the dosages of the vaccines were not observed, suggesting that standard doses are appropriate.

**What is already known on this topic**

Two vaccines against herpes zoster (shingles) are available: a live attenuated vaccine and an adjuvant recombinant subunit vaccine
No trials have directly compared the safety, efficacy, and effectiveness of these vaccines

**What this study adds**

The adjuvant recombinant subunit vaccine might prevent more cases of herpes zoster than the live attenuated vaccine
Compared with the live attenuated vaccine, however, the recombinant subunit vaccine might carry a greater risk of adverse events at injection sites

## Web extra

Extra material supplied by authors

**Supplementary information:** Appendices S1-S23 and references

## Notes

Contributors: ACT obtained funding, conceived the research, participated in pilot testing the screening and data abstraction forms, helped conceptualise the analysis, interpreted the data, and wrote the first draft of the manuscript. ACT is the guarantor. WZ coordinated the study, screened citations and full texts, resolved discrepancies, verified data accuracy and analysis, prepared the tables and figures, and helped draft sections of the manuscript. RC helped coordinate the study, screened citations and full texts, abstracted data, resolved discrepancies in risk of bias assessments, and reviewed the manuscript. AAV analysed and helped interpret the data and reviewed the manuscript. PAK, VN, MG, RW, and JPS helped to screen citations and full texts, abstracted data, and reviewed the manuscript. PAK, VN, and MG carried out risk of bias assessment. AVP provided clinical content expertise, verified categorisation of patient population health condition for analysis, and reviewed the manuscript. SES obtained funding, provided clinical content expertise, helped conceive the research, and reviewed the manuscript. The corresponding author attests that all listed authors meet authorship criteria and that no others meeting the criteria have been omitted.

Ethical approval: Not required.

Data sharing: The full dataset and statistical code are available from the corresponding author on reasonable request.

Transparency: The lead author (ACT) affirms that the manuscript is an honest, accurate, and transparent account of the study being reported; that no important aspects of the study have been omitted; and that any discrepancies from the study as planned (and, if relevant, registered) have been explained.
