# Supplementary material for: Efficacy, effectiveness, and safety of herpes zoster vaccines in adults aged 50 and older: systematic review and network meta-analysis
Source: BMJ. 2018 Oct 25;363:k4029. doi: 10.1136/bmj.k4029 (PMC6201212; doi:10.1136/bmj.k4029)
Supplement: Supplementary file 1 — Supplementary information: Appendices S1-S23 and references [file tria044865.ww1.pdf]

Contents

Appendix S1: Outcome Definitions 3

Appendix S2: Literature Search Strategy for MEDLINE, EMBASE & CENTRAL 1

Appendix S3: Grey Literature Sources 1

Appendix S4: List of Immune-Compromising Conditions 2

Appendix S5: Zero Event Studies 4

Appendix S6: Descriptive Summary of Studies Not Included in Analysis (n=5) 5

Appendix S7: Study Characteristics 7

Appendix S8: Patient and Intervention Characteristics 9

Appendix S9: Summary Results of the Cochrane Collaboration Risk of Bias Assessment (n=22 Randomized Controlled Trials) 12

Appendix S10: Summary Results of the Newcastle-Ottawa Scale Assessment (n=3 Cohort Studies, 1 Case Control Study) 13

Appendix S11: Summary Results of the Cochrane Effective Practice and Organisation of Care (EPOC) Risk of Bias Assessment (n= 1 Non-Randomized Controlled Trial) 14

Appendix S12: Transitivity Plots for All Outcomes 15

Appendix S13: Funnel Plots for Publication Bias 19

Appendix S14: Additional Results for Physician or Lab Confirmed HZ Cases: Pairwise Meta-Analyses and Network Meta-Analyses 20

Appendix S15: Additional Results for Suspected HZ Cases: Pairwise Meta-Analyses and Network Meta-Analyses 22

Appendix S16: Additional Results for HZ Ophthalmicus: Pairwise Meta-Analyses and Network Meta-Analyses 24

Appendix S17: Additional Results for Post-Herpetic Neuralgia: Pairwise Meta-Analyses and Network Meta-Analyses 25

Appendix S18: Additional Results for Injection Site Adverse Events: Pairwise Meta-Analyses and Network Meta-Analyses 26

Appendix S19: Additional Results for Systemic Adverse Events: Pairwise Meta-Analyses and Network Meta-Analyses 28

Appendix S20: Additional Results for Serious Adverse Events: Pairwise Meta-Analyses and Network Meta-Analyses 29

Appendix S21: Additional Results for Withdrawal Due to Adverse Events: Pairwise Meta-Analyses and Network Meta-Analyses 30

Appendix S22: Additional Results for Death: Pairwise Meta-Analyses and Network Meta-Analyses 31

Appendix S23: Sensitivity Analysis for Dose-Effects 33

References 34

Appendix S1: Outcome Definitions

Outcome	Definition
Confirmed herpes zoster infection	Subjects who underwent clinical diagnosis of herpes zoster infection through laboratory testing (e.g. polymerase-chain-reaction assay, virus culture) and/or examination by a physician were classified as having confirmed cases of herpes zoster infection.
Suspected herpes zoster infection	Subjects with non-injection-site herpes-zoster like rashes, varicella-like rashes, vesicular rashes or unilateral rashes (as defined by the investigators) and no other alternative clinical diagnoses were classified as having suspected cases of herpes zoster infection.
Herpes zoster ophthalmicus	Subjects with herpes zoster infection in the ocular region
Post-herpetic neuralgia	Subjects with pain continuing 90 days or longer after the onset of the shingles rash. All efficacy and effectiveness data are based on this definition and also known as PHN-90.[1]
Quality-of-life	Quality-of-life was measured by EuroQol (EQ5D), Health Utilities Index Mark 2 (HUI2), Health Utilities Index Mark 3 (HUI3) or Short Form 6 Dimensions (SF6D).
Injection site adverse events	Local reactions such as pain, redness, swelling, induration, pruritus, etc. at the injection site.
Systematic adverse events	Generalized reactions such as headache, myalgia, fever, fatigue, etc.
Serious adverse events	Any events requiring hospitalization (initial or prolonged) or medical intervention to prevent permanent damage/impairment; resulting in birth defect, disability/permanent damage, death or life-threatening condition.[2]
Withdrawal due to adverse events	The number of patients reported as withdrawn from the study due to adverse events.
Potential immune mediated diseases	A group of diseases characterized by dysregulated immune responses leading to tissue damaging inflammation
Death	The number of subjects reported for death regardless of causality.

[1] Yawn BP. Post-Shingles Neuralgia by Any Definition Is Painful, but Is It PHN? *Mayo Clinic Proceedings.* 2011;86(12):1141-1142. doi:10.4065/mcp.2011.0724.

[2] US Food and Drug Administration. What is a Serious Adverse Event? [updated February 1, 206]. Available from https://www.fda.gov/Safety/MedWatch/HowToReport/ucm053087.htm [accessed January 10, 2018.

Appendix S2: Literature Search Strategy for MEDLINE, EMBASE & CENTRAL

EBM Reviews - Cochrane Central Register of Controlled Trials January 2017
Embase 1974 to 2017 January 19
Epub Ahead of Print, In-Process & Other Non-Indexed Citations, Ovid MEDLINE(R) Daily and Ovid MEDLINE(R) 1946 to Present

1. Herpes Zoster Vaccine/
2. exp Vaccines/
3. exp Herpes Zoster/
4. Vaccination/
5. Neuralgia, Postherpetic/
6. exp Immunization/
7. Encephalitis, Varicella Zoster/ or Herpesvirus 3, Human/
8. (vaccine* or vaccinat* or immuniz* or inocul*).tw.
9. (zoster or shingles or herpes-zoster or varicella-zoster or postherpetic or post-herpetic or hhv-3 or hhv3 or herpesvirus 3).tw.
10. 3 or 5 or 7 or 9
11. 2 or 4 or 6 or 8
12. 10 and 11
13. 1 or 12
14. zostavax.tw.
15. Shingrix.tw.
16. "HZ/su".tw.
17. 13 or 14 or 15 or 16
18. exp Animals/ not (exp Animals/ and Humans/)
19. 17 not 18
20. antibody formation/ or seroconversion/
21. Antibodies, Viral/
22. (Geometric mean adj2 (titre* or titer* or concentrat* or rise*)).tw.
23. (antibod* or seroconver*).tw.
24. (Immunogenic* or titre* or titer*).tw.
25. enzyme-linked immunosorbent assay/ or enzyme-linked immunospot assay/
26. (enzyme-linked immunosorbent assay or enzyme-linked immunospot assay or ELISA or ELISPOT).tw.
27. (T cell or CD4 or CD8 or B cell or cytokine).tw.
28. b-lymphocytes/ or t-lymphocytes/ or cd4-positive t-lymphocytes/ or cd8-positive t-lymphocytes/
29. Cytokines/
30. (seropositiv* or seronegativ* or gmt or gmc or gmfr).tw.
31. or/20-30
32. 19 and 31
33. 19 use ppez
34. **32 use ppez**
35. varicella zoster vaccine/
36. vaccine/
37. exp Herpes Zoster/
38. Vaccination/

39. postherpetic neuralgia/
40. exp Immunization/
41. exp Varicella zoster virus/
42. (vaccine* or vaccinat* or immuniz* or inocul*).tw.
43. (zoster or shingles or herpes-zoster or varicella-zoster or postherpetic or post-herpetic or hhv-3 or hhv3 or herpesvirus 3).tw.
44. 37 or 39 or 41 or 43
45. 36 or 38 or 40 or 42
46. 44 and 45
47. 35 or 46
48. zostavax.tw.
49. Shingrix.tw.
50. "HZ/su".tw.
51. 47 or 48 or 49 or 50
52. exp animal/ not (exp animal/ and human/)
53. 51 not 52
54. immunogenicity/ or seroconversion/
55. antibody/ or virus antibody/
56. (Geometric mean adj2 (titre* or titer* or concentrat* or rise*)).tw.
57. (antibod* or seroconver*).tw.
58. (Immunogenic* or titre* or titer*).tw.
59. enzyme linked immunosorbent assay/ or enzyme linked immunospot assay/
60. (enzyme-linked immunosorbent assay or enzyme-linked immunospot assay or ELISA or ELISPOT).tw.
61. (T cell or CD4 or CD8 or B cell or cytokine).tw.
62. B lymphocyte/ or t lymphocyte/ or cd4+ t lymphocyte/ or cd8+ t lymphocyte/
63. Cytokine/
64. (seropositiv* or seronegativ* or gmt or gmc or gmfr).tw.
65. or/54-64
66. 53 and 65
67. random*.tw.
68. clinical trial*.mp.
69. exp treatment outcome/
70. or/67-69
71. exp cohort analysis/
72. exp longitudinal study/
73. exp prospective study/
74. exp follow up/
75. cohort*.tw.
76. exp case control study/
77. (case* and control*).tw.
78. or/71-77
79. (Ae or to or co).fs.
80. exp ADVERSE DRUG REACTION/
81. exp SIDE-EFFECT/
82. risk/
83. ((adverse adj3 (effect* or event* or reaction*)) or side effect* or complication* or risk*).tw.
84. postmarketing surveillance/

85. or/79-84
86. 70 or 78 or 85
87. 53 and 86
88. 87 use oemezd
89. 66 use oemezd
90. 19 use cctr
91. 32 use cctr
92. 87 use cctr
93. 66 use cctr
94. 90 or 92
95. 91 or 93
96. 33 or 88 or 94
97. limit 96 to yr="2000 -Current"
98. remove duplicates from 97
99. 34 or 89 or 95
100. remove duplicates from 99
101. limit 96 to yr="1946 - 1999"
102. remove duplicates from 101
103. 98 or 102
104. remove duplicates from 103

Appendix S3: Grey Literature Sources

1. **Trial registries:**
    - Biomed Central. ISRCTN Registry: http://www.isrctn.com/
    - National Institute of Medical Statistics, Indian Council of Medical Research. Clinical Trials Registry - India (CTRI): http://ctri.nic.in/Clinicaltrials/advancesearchmain.php
    - US National Institutes of Health. ClinicalTrials.gov: http://clinicaltrials.gov/ct/screen/AdvancedSearch
    - Thomson CenterWatch. CenterWatch Clinical Trials Listing Service: http://www.centerwatch.com/clinical-trials/listings/

2) General grey databases:

- Government of Canada: http://publications.gc.ca/site/eng/search/eCollection.html
- GreyNet International: http://www.greylit.org
- SIGLE (System for Information on Grey Literature in Europe): http://www.opengrey.eu

3) International databases:

- Agency for Healthcare Research and Quality: http://www.ahrq.gov/research/index.html
- LILACS - Latin-American and Caribbean Center on Health Sciences Information: http://lilacs.bvsalud.org/en/
- WHO (WHOLIS): http://dosei.who.int/uhtbin/cgisirsi/Tue+Apr++5+17:45:43+MEST+2016/0/49

4) Theses and dissertations:

- Center for Research Libraries Foreign Dissertation: https://www.crl.edu/collections/topics/dissertations
- DART-Europe E-theses Portal: http://www.dart-europe.eu/basic-search.php
- Electronic Theses Online Service (ETHOS) | British Library: http://ethos.bl.uk/Home.do;jsessionid=D96E9CF245B0FE0199DDDB94FF4BD2A7
- Open access dissertations: https://oatd.org
- Thesis Canada Portal: http://www.bac-lac.gc.ca/eng/services/theses/Pages/theses-canada.aspx

Appendix S4: List of Immune-Compromising Conditions

Immune-compromising Condition	ICD-10 Code
Congenital (primary) immunodeficiency	
Cellular immune deficiencies:<br>*T cell, natural killer T cell, mixed cellular and antibody defects, severe combined immune deficiency (SCID)*	D80 Immunodeficiency with predominantly antibody defects<br>D81 Combined Immunodeficiencies<br>D82 Immunodeficiency associated with other major defects<br>D83 Common variable immunodeficiency<br>D84 Other immunodeficiencies
Acquired (secondary) immunodeficiency	
Malignant Hematologic Disorders:<br>*Blood dyscrasia, leukemia, lymphoma, other malignant neoplasms affecting bone marrow or lymphatic systems*	C81 Hodgkin lymphoma<br>C82 Follicular lymphoma<br>C83 Non-follicular lymphoma<br>C84 Mature T/NK-cell lymphomas<br>C85 Other and unspecified types of non-Hodgkin lymphoma<br>C86 Other specified types of T/NK-cell lymphomas<br>C90 Multiple myeloma and malignant plasma cell neoplasms<br>C91 Lymphoid leukemia<br>C92 Myeloid leukemia<br>C93 Monocytic leukemia<br>C94 Other leukemias of specified cell type<br>C95 Leukemia of unspecified cell type<br>C96 Other and unspecified malignant neoplasms of lymphoid, hematopoietic and related tissue<br>D75.9 Disease of blood and blood-forming organs, unspecified
Human Immunodeficiency Virus (HIV) Infection	B20 HIV disease resulting in infectious and parasitic disease<br>B21 HIV disease resulting in malignant neoplasms<br>B22 HIV disease resulting in other specific diseases<br>B23 HIV disease resulting in other conditions<br>B24 Unspecified HIV disease
Post-Solid Organ Transplantation	Z94 Transplanted organ and tissue status
Post-Hematopoietic Stem Cell Transplantation	Z94.8 Other transplanted organ and tissue status
Long-term immunosuppressive therapy:<br>1. Steroids<br>*Does not include: inhaled, topical, locally injected steroids; maintenance replacement therapy for adrenal insufficiency; short-term (<14 days); low dose (<2mg/kg/day for a child or <20mg/day of prednisone for adult (>10kg)); long-term alternate-day treatment with short-acting preparations*<br>2. Cancer Chemotherapy<br>(up to 3 months post-therapy)	*Not applicable*

Does not include tamoxifen, hydroxyurea, gonadotropin release inhibitors<br>3. Radiation therapy<br>4. Monoclonal Antibodies:<br>*Rituximab, infliximab, adalimumab, alemtuzumab, basiliximab*<br>*Anti-TNFs should be considered on case-by-case basis*<br>Other Immunosuppressants:<br>*6-mercaptopurine >1.5mg/kg/day, anti-thymocyte globulin, azathioprine >3mg/kg/day, cyclophosphamide, cyclosporine, leflunomide, methotrexate >0.4mg/kg/day, mitoxantrone, mycophenolate mofetil, sirolimus, tacrolimus*	

Appendix S5: Zero Event Studies

Outcomes	# of studies with zero events excluded from the main analysis	# of studies with zero events excluded from the dose-effects analysis
Lab or Physician Confirmed HZ cases	3[1-3]	6[1-6]
Suspected HZ cases	3[1-3]	3[1-3]
HZ ophthalmicus	None	None
Post-herpetic neuralgia	None	None
Quality-of-life	None	None
Injection site AEs	None	None
Systemic AEs	None	None
Serious AEs	4[1-3, 7]	6[1-4, 6, 7]
Withdrawal due to AEs	4[2, 7-9]	7[2-4, 6-9]
Potential immune mediated diseases	None	None
Death	5[1, 3, 7, 9, 10]	8[1, 3, 4, 6, 7, 9-11]

Abbreviations: AE – adverse event; HZ - herpes zoster

Appendix S6: Descriptive Summary of Studies Not Included in Analysis (n=5)

First Author, Year	Study Design	Study Location(s)	# Study Centres	Study Period	Study Length (mos.)	Trial Arms	Summar of findings
Diez-Domingo, 2015[12]	RCT	Germany, Spain	Multi	2011-2013	25	ZVL<br>≥19,400 PFU (1 dose, IM)<br>vs.<br>ZVL<br>≥19,400 PFU (1 dose, SC)	*Safety*<br>• Injection-site AEs were less frequent with IM than SC route: 34% (60/177) vs 64% (114/177)<br>• Systemic AEs were similar between IM & SC: 23% (41/177) vs. 23% (40/177)<br>• Three subjects experienced serious AEs: 1(IM) vs. 2(SC)<br>*Efficacy*<br>• One subject in the IM group reported a zoster-like rash (right thoracic dermatome)
Giderman, 2008[13]	RCT	USA	Multi	2005	3	ZVL 50000PFU (refrigerated, 1 dose)<br>vs.<br>ZVL 50000PFU (Frozen, 1 dose)	*Safety*<br>• Injection-site AEs were less frequent in the refrigerated than frozen group: 36% (64/180) vs. 46% (85/183)<br>• Systemic AEs were similar between refrigerated & frozen group: 19% (34/180) vs. 21% (39/183)<br>• One subject experienced serious AEs in the refrigerated group<br>*Efficacy*<br>• One subject in the refrigerated group reported non-injection-site varicella-like rash with three lesions
GlaxoSmithKline, 2016[14]	RCT	Estonia, USA	Multi	2013-2015	4	HZ/su<br>(2 doses. 2 months)<br>vs.<br>HZ/su<br>(2 doses, 6 months)<br>vs.<br>HZ/su	*Safety*<br>• Injection-site AEs and systemic AEs were similar across the trial arms<br>• Two subjects reported serious AEs: 1(2 month) vs. 1(12 month)<br>*Efficacy*<br>• Not assessed

						(2 doses, 12 months)	
Vesikari, 2013[15]	RCT	Finland, Germany, Italy, Spain, Netherlands	Multi	2007-2009	23	ZVL ≥19,400 PFU (1 dose) vs. ZVL ≥19,400 PFU (2 doses, 1 month) vs. ZVL ≥19,400 PFU (2 doses, 3 month)	*Safety*<br>• Injection-site AEs were similar between 1 month vs. 3 month schedule: 42% (98/232) vs. 42% (94/221)<br>• Systemic AEs were similar between 1 month vs. 3 month: 21% (48/232) vs. 15% (34/221)<br>• Four subjects reported *serious* AEs: 2 (1 month) vs. 2(3 months)<br>*Efficacy*<br>• One subject in each of 1 month and 3 month group reported varicella-like rash
Vink, 2016[16]	RCT	Japan	Single	2013-2014	17	HZ/su 50µg (2 doses, SC)<br>vs.<br>HZ/su 50µg (2 doses) IM	*Safety*<br>• Injection-site AEs were more frequent in SC group compared to IM group<br>• Systemic AE were similar between SC and IM group<br>• Three subjects reported serious AEs: 1(IM) vs. 2(SC)<br>*Efficacy*<br>• No HZ cases were reported during the study period

Abbreviations: AE – adverse event; HZ - herpes zoster; HZ/su – adjuvant, recombinant subunit herpes zoster vaccine; IM – intramuscular; SC – subcutaneous; ZVL – live-attenuated herpes zoster vaccine

Appendix S7: Study Characteristics

First Author, Year	Trial Registry Identifier	Funding Source Type	Study Design	Single / Multi centre	Study Location(s)	# Study Centres	Study Period	Study Length (mos.)	Outcomes reported*
Beals, 2016[4]	NCT01385566	Industry	RCT	Multi	USA	4	2011-2013	20	Safety, HZ
Berger, 1998[5]	NR	Industry	RCT	NR	Switzerland	NR	NR	NR	Safety, HZ
Chlibek, 2013[1]	NCT0080246 EudraCT2008-005120-86	Industry	RCT	Multi	Czech Republic, Spain, USA	12	2009-2010	18	Safety, HZ
Chlibek, 2014[17, 18]	NCT00434577	Industry	RCT	Multi	Czech Republic, Germany, Sweden, Netherlands	11	2007-2011	48	Safety
Cunningham, 2016[19, 20]	NCT01165177 NCT01165229	Industry	RCT	Multi	NR	215	2010-2015	60	Safety, HZ, HZO, PHN
Diez-Domingo, 2015[12]	NCT01391546 EudraCT 2009-012458-19	Industry	RCT	Multi	Germany, Spain	10	2011-2013	25	Safety, HZ
Gilderman, 2008[13]	NR	Industry	RCT	Multi	USA	NR	2005	3	Safety, HZ
GlaxoSmithKline, 2016[14]	NCT01751165	Industry	RCT	Multi	Estonia, USA	4	2013-2015	25	Safety
Hata, 2016[2]	UMIN000004771	Public	RCT	Single	Japan	1	2011-2014	34	Safety, HZ
Kerzner, 2007[9]	NR	Industry	RCT	Multi	USA, Germany, UK, Italy, Netherlands	20	2005-2006	6	Safety, HZ
Lal, 2015[8, 20]	NCT01165177	Industry	RCT	Multi	NR	213	2010-2014	45	Safety, HZ
Langan, 2013[21, 22]	NR	Public	Cohort	NR	USA	NR	2007-2009	36	HZ, PHN
Leroux-Roels, 2012[6]	NCT00492648	Industry	RCT	Single	Belgium	1	2004-2008	42	Safety, HZ
Levin, 2016[23]	NCT01245751	Industry	NRCT	NR	USA	NR	2011-2015	49	Safety
MacIntyre, 2010[10]	NCT00535730	Industry	RCT	Multi	Australia, Canada, Germany, Italy, Spain, UK	18	2007-2008	8	Safety, HZ
Marin, 2015[24]	NR	Public	Case-control	Multi	USA	3	2010-2011	21	HZ, PHN
Merck & Co, 2015[7]	NCT00886613	Industry	RCT	NR	USA	NR	2009	9	Safety
Murray, 2011[25]	NCT00550745	Industry	RCT	Multi	Canada, Germany, Spain, UK, USA	46	2007-2009	16	Safety

Oxman, 2005[26-33]	NCT00007501	Industry & public	RCT	Multi	USA	22	1998-2004	65	Safety, HZ, PHN
Russell, 2015[34]	NCT00546819	Industry	RCT	Multi	NR	45	2007-2010	34	Safety, HZ, HZO
Schmader, 2012[35, 36]	NCT00534248	Industry	RCT	Multi	NR	105	2007-2010	27	Safety, HZ
Tseng, 2016[37-41]	NR	Public	Cohort	Multi	USA	NR	2007-2015	102	HZ, HZO
Tyring, 2007[11]	NR	NR	RCT	Multi	USA, Canada, UK, Germany, Belgium	18	2003-2004	8	Safety, HZ
Vermeulen, 2012[3]	NCT00109122	Industry	RCT	Multi	USA, Netherlands	6	2001-2003	15	Safety, HZ
Vesikari, 2013[15, 42]	NCT00561080 EudraCT 2007-000744-28	Industry	RCT	Multi	Finland, Germany, Italy, Spain, Netherlands	32	2007-2009	23	Safety, HZ
Vink, 2017[16, 43]	NCT01777321	Industry	RCT	Single	Japan	1	2013-2014	17	Safety, HZ
Zhang, 2012[44, 45]	NR	Public	Cohort	Multi	USA	NR	2006-2009	48	HZ

** not all could be included in the pooled analyses*

Abbreviations: HZ – herpes zoster; HZO – hepes zoster ophthalmicus; PHN – postherpetic neuralgia; NR – not reported; RCT – randomized controlled trials; UK – United Kingdom; USA – United States of America

Appendix S8: Patient and Intervention Characteristics

First author, Year	History of HZ infection	History of chicken pox	Health status	Study N	% Female	Patient age	Trial arms
Beals, 2016[4]	No	Yes	Immunocompetent *(uncertain if subjects on steroids were excluded)*	223	56.0	Mean: 60.8 (SD 7.9)	ZVL (Full dose, SC) vs Zostavax (1/3 dose, SC) vs ZVL (Full dose, ID) vs Zostavax (1/3 dose, ID) vs ZVL (1/10 dose, ID) vs ZVL (1/27 dose, ID)
Berger, 1998[5]	No	Yes	Immunocompetent *(uncertain if subjects on steroids were excluded)*	200	41.0	Range: 55-88	ZVL 3200PFU (1 dose) vs ZVL 8500PFU (1 dose) vs ZVL 41650PFU (1 dose)
Chlibek, 2013[1]	No	Yes	Immunocompetent *(subjects receiving steroids at ≥0.5 mg/kg for less than 14 days were included)*	188	56.6	NR	HZ/su 50µg (2 doses) vs Placebo (2 doses)
Chlibek, 2014[17, 18]	No	Yes	Immunocompetent	660	52.9	NR	HZ/su 25µg (2 doses) vs HZ/su 50µg (2 doses) vs HZ/su 100µg (2 doses) vs HZ/su 100µg (1 dose) vs
Cunningham, 2016[19, 20]	No	NR	Immunocompetent	14,816	54.9	Mean: 75.6 (SD: 4.7)	HZ/su 50µg (2 doses) vs Placebo (2 doses)
Diez-Domingo, 2015[12]	No	Yes	Immunocompetent *(uncertain if subjects on steroids were excluded)*	354	55.1	Mean: 62.6 (SD: 8.4)	ZVL ≥19,400 PFU (1 dose, IM) vs ZVL ≥19,400 PFU (1 dose, SC)
Gilderman, 2008[13]	No	Yes	Immunocompetent *(uncertain if subjects on steroids were excluded)*	367	55.0	NR	ZVL 50000PFU

							(refrigerated, 1 dose) vs ZVL 50000PFU (Frozen, 1 dose)
GlaxoSmithKline, 2016[14]	No	Yes	Immunocompetent<br><br>*(uncertain if subjects on steroids were excluded)*	354	69.5	Mean: 64.2 (SD 8.9)	HZ/su (2 doses. 2 months) vs HZ/su (2 doses, 6 months) vs HZ/su (2 doses, 12 months)
Hata, 2016[2]	No	NR	Immunocompetent	54	44.4	Mean: 66.2 (SD 4.4)	ZVL 50000PFU (1 dose) vs Placebo (1 dose)
Kerzner, 2007[9]	No	Yes	Immunocompetent<br><br>*(uncertain if subjects on steroids were excluded)*	762	56.0	NR	ZVL 58000PFU vs Placebo
Lal, 2015[8, 20]	No	NR	Immunocompetent	15,411	61.2	Mean: 62.3 (SD: 9.0)	HZ/su 50µg (2 doses) vs Placebo (2 doses)
Langan, 2013[21, 22]	No	NR	Mixed	766,330	67.7	NR	ZVL vs Unvaccinated
Leroux-Roels, 2012[6]	No	No	Immunocompetent<br><br>*(subjects receiving steroids at ≥0.5 mg/kg for less than 14 days were included)*	135	68.2	NR	HZ/su 50µg (2 doses) vs Varilrix (2 doses) vs HZ/su 50µg + Varilrix (2 doses)
Levin, 2016[23]	No	Yes	Immunocompetent<br><br>*(uncertain if subjects on steroids were excluded)*	600	57.2	Mean: 71.1	ZVL booster (1 dose) vs ZVL (1 dose)
MacIntyre, 2010[10]	No	Yes	Immunocompetent<br><br>*(uncertain if subjects on steroids were excluded)*	473	57.7	Mean: 66.2 (SD 5.6)	ZVL+ PNEUMOVAX 23 (1 dose) vs Placebo + PPV23 (1 dose)
Marin, 2015[24]	Yes	NR	Mixed	628	61.5	NR	ZVL vs Unvaccinated
Merck & Co, 2015[7]	No	Yes	Immunocompetent<br><br>*(uncertain if subjects on steroids were excluded)*	79	61.7	NR	ZVL (2 doses) vs Placebo (2 doses)

Murray, 2011[25]	NR	NR	Immunocompetent *(does not define if subjects on immunosuppressants were excluded)*	11,999	41.0	NR	ZVL (1 dose) vs Placebo (1 dose)
Oxman, 2005[26-33]	NR	Yes	Immunocompetent	38,546	41.0	Median: 69.0	ZVL 18700PFU-60000PFU (1 dose) vs Placebo (1 dose)
Russell, 2015[34]	No	Yes	Immunocompromised	309	41.0	NR	ZVL (1 dose) vs Placebo (1 dose)
Schmader, 2012[35, 36]	No	Yes	Immunocompetent	22,439	61.9	NR	ZVL (1 dose) vs Placebo (1 dose)
Tseng, 2016[37-41]	No	NR	Immunocompetent *(uncertain if subjects on steroids were excluded)*	704,312	53.6	Mean: 68.7 (SD 7.7)	ZVL vs Unvaccinate d
Tyring, 2007[11]	No	Yes	Immunocompetent *(uncertain if subjects on steroids were excluded)*	698	59.9	NR	ZVL 207000PFU (1 dose) vs ZVL 58000PFU (1 dose)
Vermeulen, 2012[3]	No	Yes	Immunocompetent	210	63.2	NR	ZVL 23000PFU (1 dose) vs Placebo (1 dose)
Vesikari, 2013[15, 42]	No	Yes	Immunocompetent *(uncertain if subjects on steroids were excluded)*	759	55.5	Mean: 76.1	ZVL (1 dose) vs ZVL (2 doses, 1 month) vs ZVL (2 doses, 3 month)
Vink, 2017[16, 43]	No	Yes	Immunocompetent *(uncertain if subjects on steroids were excluded)*	60	50.0	Mean: 61.9 (SD 7.7)	HZ/su 50µg (2 doses, SC) vs HZ/su 50µg (2 doses) IM
Zhang, 2012[44, 45]	No	NR	Immunocompromised	463,541	72.3	Mean: 74.0 (SD 8.0)	ZVL vs Unvaccinate d

Abbreviations: ID – intradermal; HZ – herpes zoster; HZ/su – adjuvant, recombinant subunit herpes zoster vaccine; IM – intramuscular; NR – not reported; SC – subcutaneous; SD – standard deviation, ZVL – live-attenuated herpes zoster vaccine

Appendix S9: Summary Results of the Cochrane Collaboration Risk of Bias Assessment (n=22 Randomized Controlled Trials)

Study ID	Random sequence generation	Allocation concealment	Blinding of participants & personnel	Blinding of outcome assessment	Incomplete outcome data	Selective reporting	Other bias
Beals 2016[4]	Low	Unclear	High	Unclear	Low	Low	High
Berger 1998[5]	Unclear	High	Unclear	Unclear	Unclear	Unclear	High
Chlibek 2013[1]	Unclear	Low	Low	Low	Low	Low	High
Chlibek 2014[17, 18]	Unclear	Unclear	Unclear	High	Low	Low	High
Cunningham 2016[19, 20]	Low	Low	Low	Low	Low	Low	High
Diez-Domingo 2015[12]	Low	Low	High	High	Low	Low	High
Gilderman 2008[13]	Unclear	Unclear	Low	Unclear	Low	Unclear	High
GlaxoSmithKline 2016[14]	Unclear	Unclear	High	High	Low	Low	High
Hata 2016[2]	Low	Low	Low	Unclear	Low	Low	Low
Kerzner 2007[9]	Unclear	Unclear	Low	Unclear	Low	Unclear	High
Lal 2015[8, 20]	Low	Unclear	Low	Low	High	Low	High
Leroux-Roels 2012[6]	Unclear	Unclear	High	High	Low	Low	High
Murray 2011[25]	Unclear	Low	Low	Low	Low	Low	High
Oxman 2005[26-33]	Unclear	Unclear	Low	Low	Low	Low	High
Russell 2015[34]	Low	Unclear	Low	Low	Low	Low	High
Schmader 2012[35, 36]	Low	Low	Low	Low	Low	Low	High
Tyring 2007[11]	Unclear	Unclear	Low	Low	Low	Unclear	High
Vermeulen 2012[3]	Low	Low	Low	Low	Low	Low	High
Vesikari 2013[15, 42]	Low	Unclear	High	High	Low	Low	High
Vink 2016[16, 43]	Unclear	Unclear	High	High	Low	Low	High
MacIntyre 2010[10]	Unclear	Unclear	Low	Low	Low	Low	High
Merck & Co 2015[7]	Unclear	Unclear	Low	Unclear	Low	Low	High

Appendix S10: Summary Results of the Newcastle-Ottawa Scale Assessment (n=3 Cohort Studies, 1 Case Control Study)

Study ID	Representativeness of the exposed cohort	Selection of the non-exposed cohort	Ascertainment of exposure	Demonstration that outcome of interest was not present at start of study	Comparability of cohorts on the basis of the design or analysis	Assessment of outcome	Adequacy of follow up of cohorts
Marin 2015[24]	Moderate	Low	Low	Low	Low	Moderate	Unclear
Langan 2013[21, 22]	Low	Low	Low	Low	Moderate	Low	Low
Tseng 2016[37-41]	Moderate	Low	Low	Low	Moderate	Low	Low
Zhang 2012[44, 45]	High	Low	Low	Low	Moderate	Low	Low

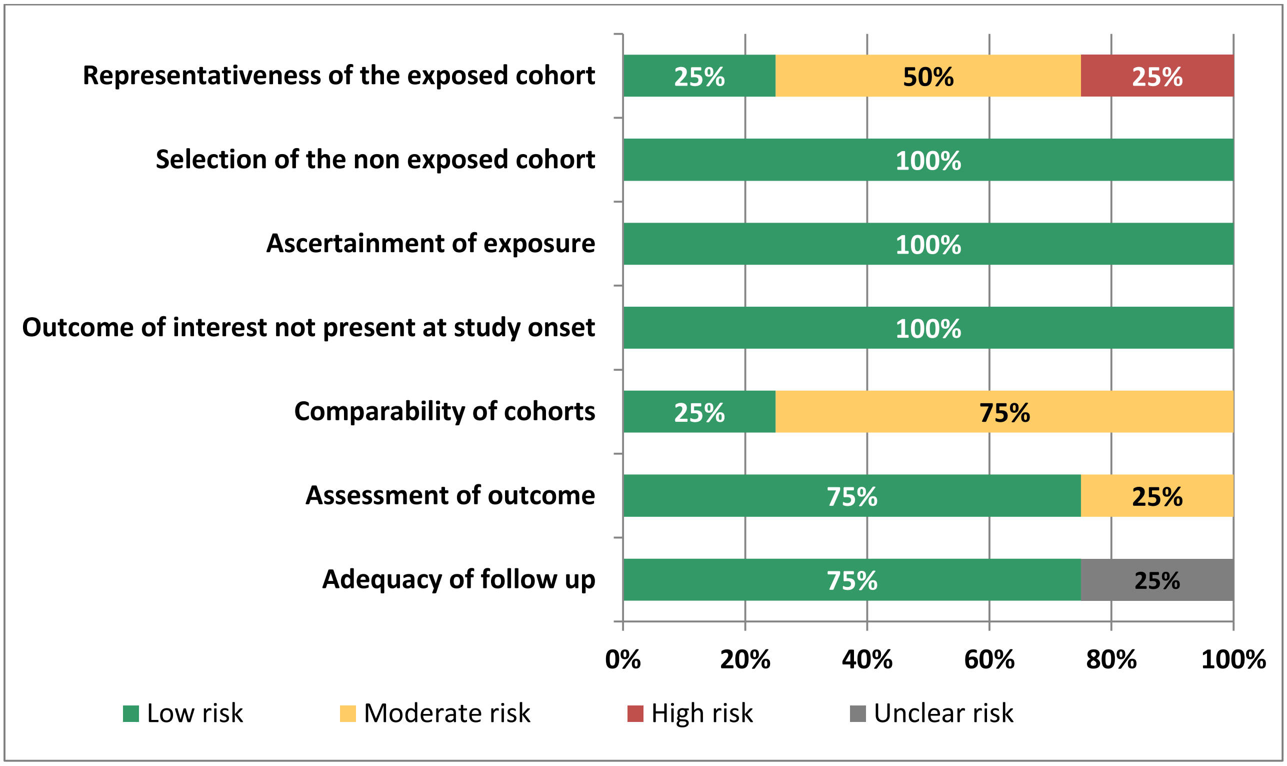

Appendix S11: Summary Results of the Cochrane Effective Practice and Organisation of Care (EPOC) Risk of Bias Assessment (n= 1 Non-Randomized Controlled Trial)

Study ID	Random sequence generation	Allocation concealment	Similar baseline outcome measures	Similar baseline characteristics	Incomplete outcome data	Blinding	Contamination	Selective outcome reporting	Other bias
Levin 2016[23]	Unclear	Unclear	Low	Low	Low	Unclear	Unclear	Low	High

Appendix S12: Transitivity Plots for All Outcomes

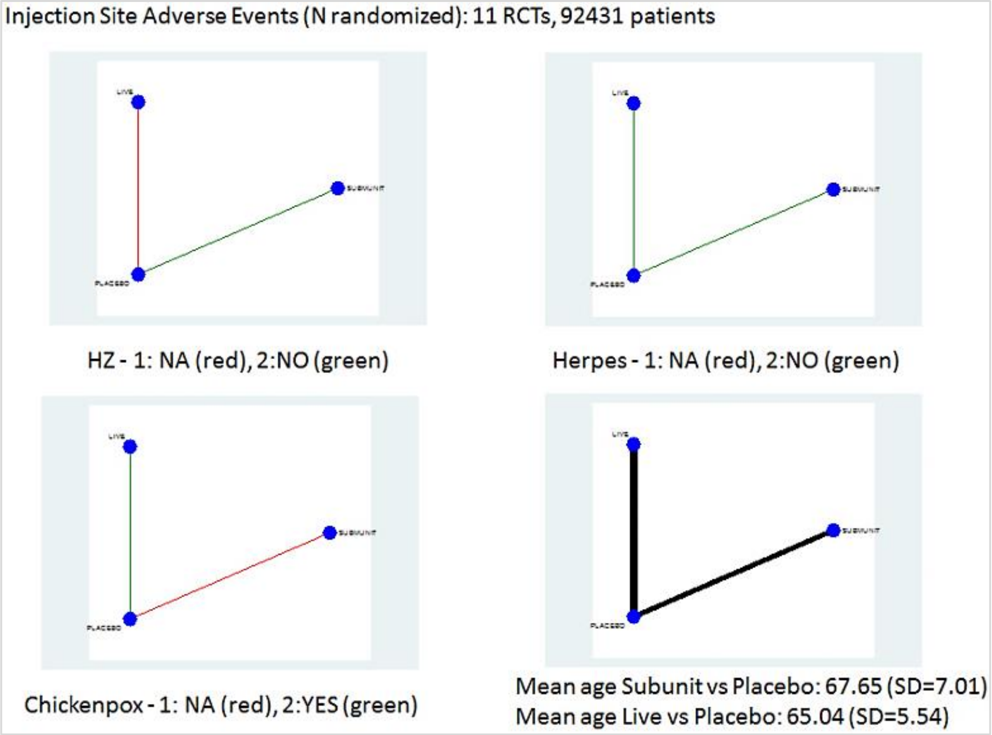

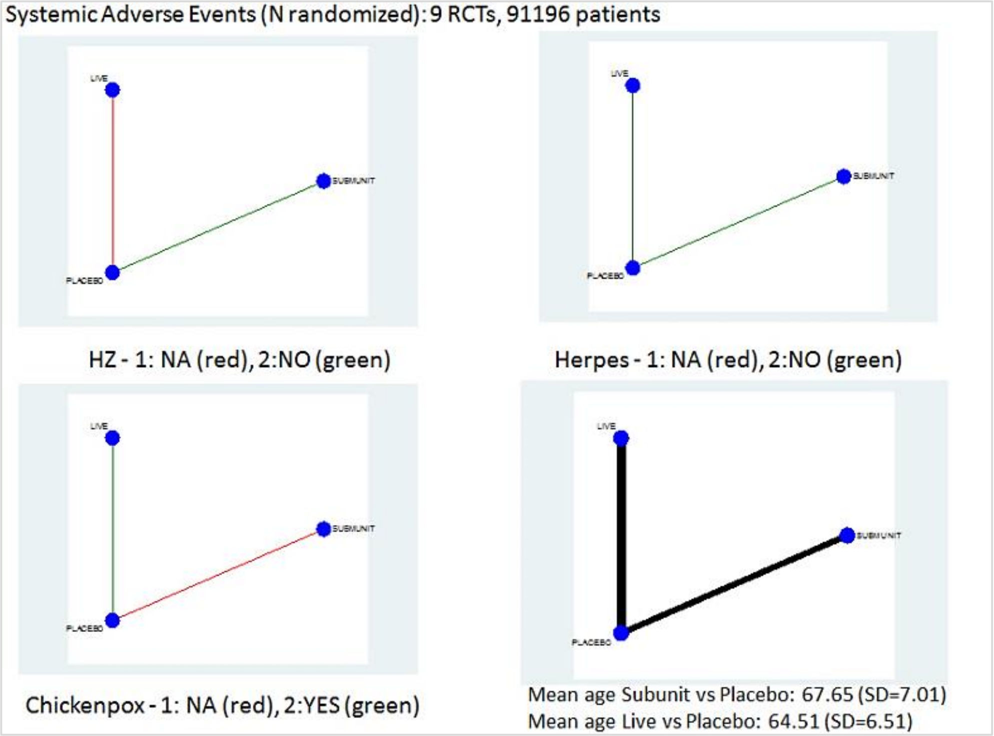

Serious Adverse Events (N randomized): 8 RCTs, 103899 patients

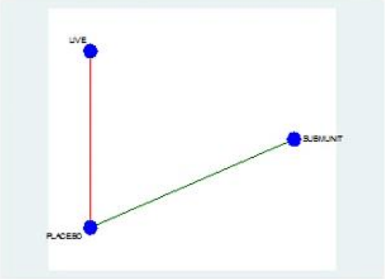

HZ - 1: NA (red), 2:NO (green)

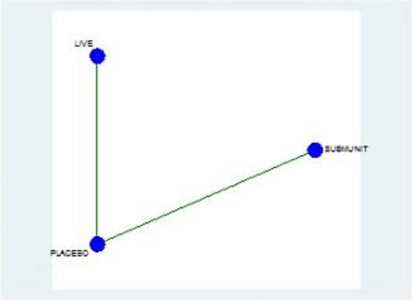

Herpes - 1: NA (red), 2:NO (green)

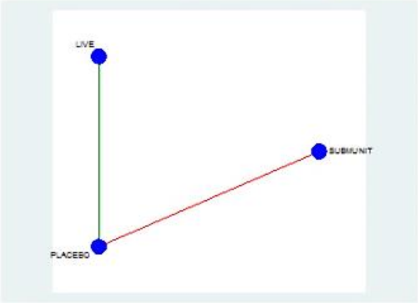

Chickenpox - 1: NA (red), 2:YES (green)

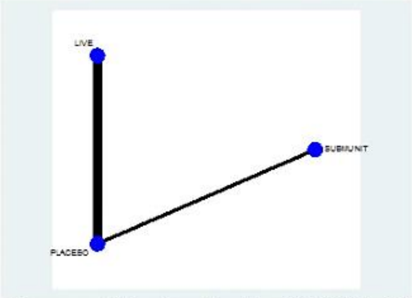

Mean age Subunit vs Placebo: 68.98 (SD=9.37)
Mean age Live vs Placebo: 64.96 (SD=6.32)

Withdrawal Due to Adverse Events (N randomized): 6 RCTs, 35678 patients

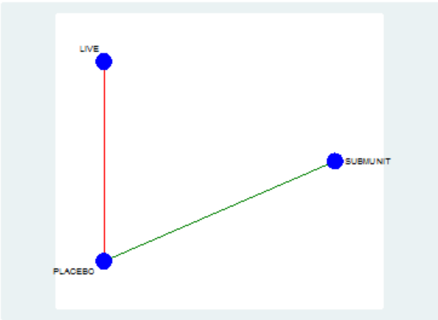

HZ - 1: NA (red), 2:NO (green)

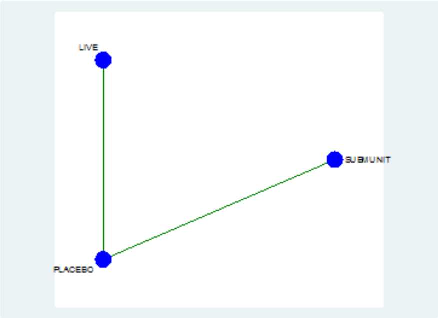

Herpes - 1: NA (red), 2:NO (green)

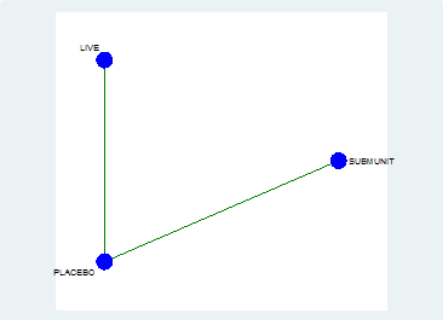

Chickenpox - 1: NA (red), 2:YES (green)

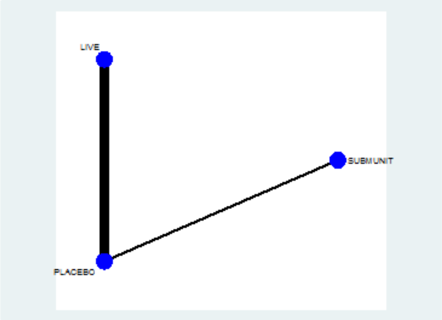

Mean age Subunit vs Placebo: 65 (SD=NA) [1 obs]
Mean age Live vs Placebo: 67.2 (SD=7.66)

Death (N randomized): 7 RCTs, 102718 patients

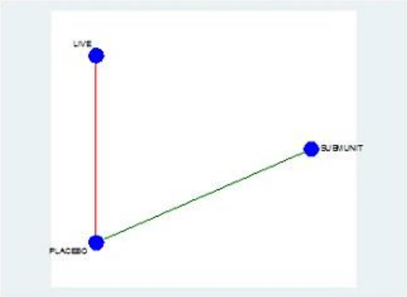

HZ - 1: NA (red), 2:NO (green)

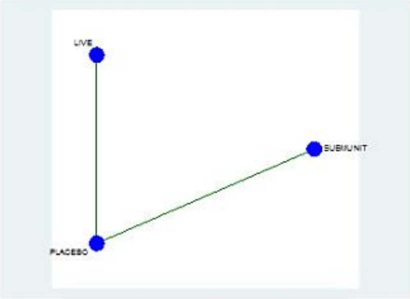

Herpes - 1: NA (red), 2:NO (green)

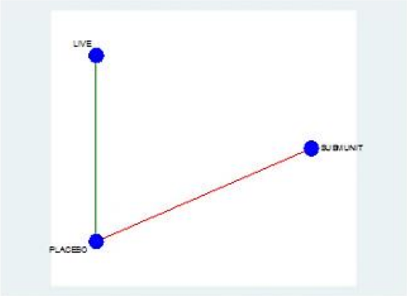

Chickenpox - 1: NA (red), 2:YES (green)

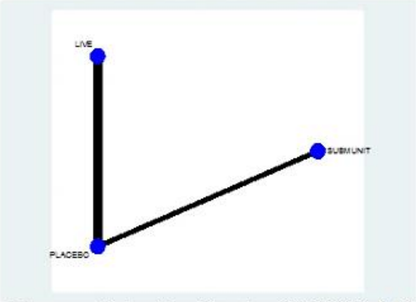

Mean age Subunit vs Placebo: 68.98 (SD=9.37)
Mean age Live vs Placebo: 65.35 (SD=7.24)

HZ_confirmed (N randomized): 5 RCTs and 1 case-control, 91230 patients

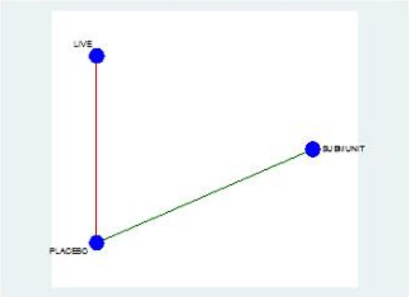

HZ - 1: NA (red), 2:NO (green)

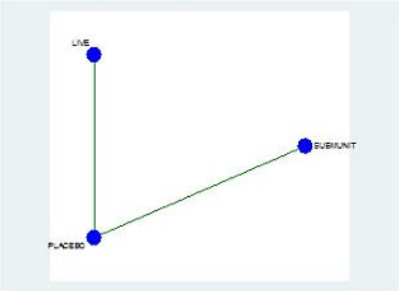

Herpes - 1: NA (red), 2:NO (green)

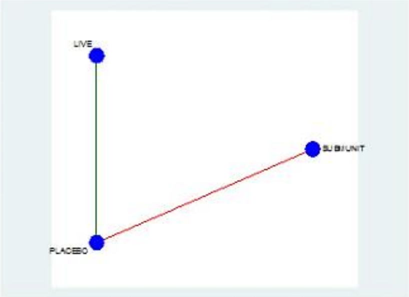

Chickenpox - 1: NA (red), 2:YES (green)

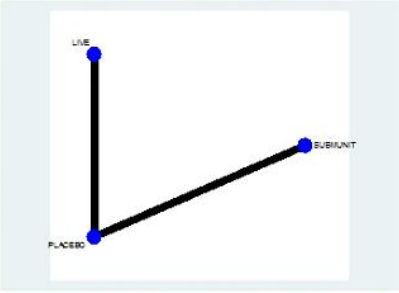

Mean age Subunit vs Placebo: 68.98 (SD=9.37)
Mean age Live vs Placebo: 62.35 (SD=10.61)

HZ_suspected (N randomized): 7 RCTs, 3 Cohort studies, 1 case-control, 2026648 patients

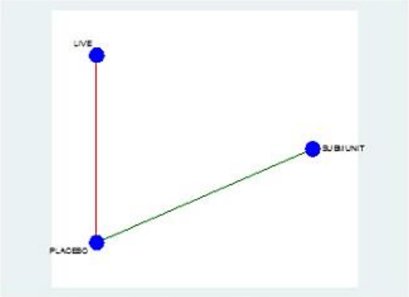

HZ - 1: NA (red), 2:NO (green)

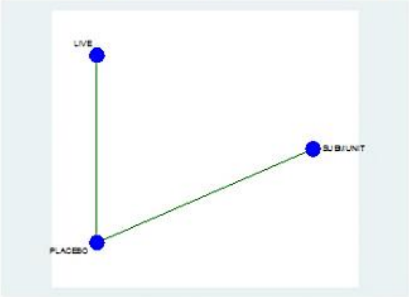

Herpes - 1: NA (red), 2:NO (green)

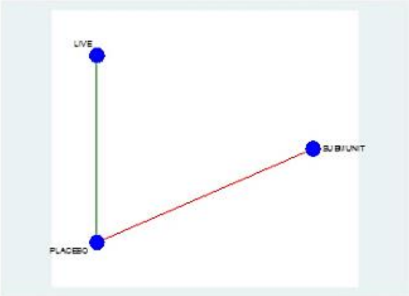

Chickenpox - 1: NA (red), 2:YES (green)

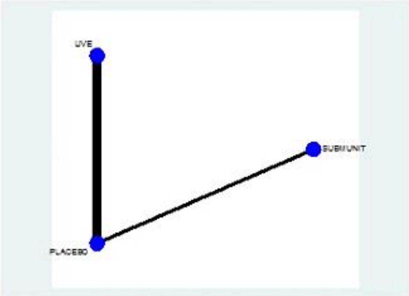

Mean age Subunit vs Placebo: 68.98 (SD=9.37)
Mean age Live vs Placebo: 64.61 (SD=5.98)

PHN (N randomized): 2 RCTs, 1 cohort study, 1 case-control, 819401 patients

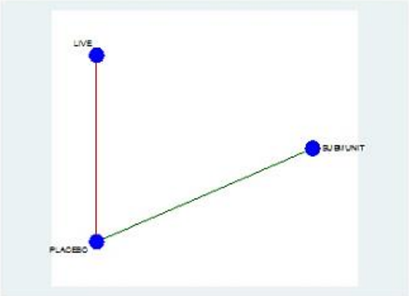

HZ - 1: NA (red), 2:NO (green)

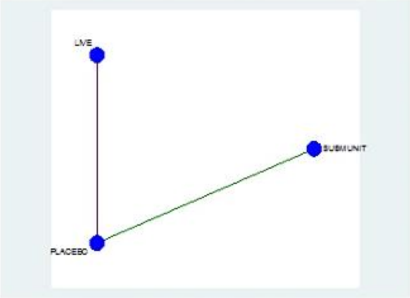

Herpes - 1: NA (red), 2:NO (green), 3:YES (purple)

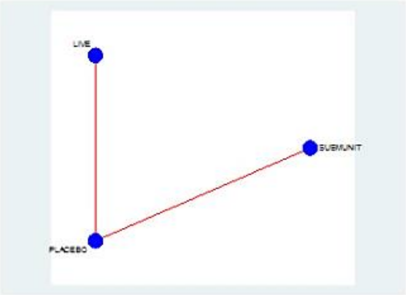

Chickenpox - 1: NA (red), 2:YES (green)

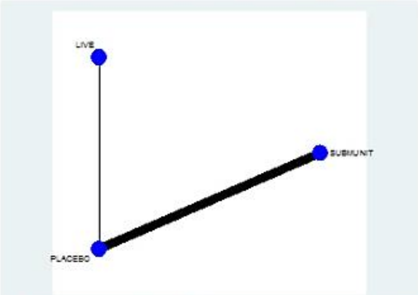

Mean age Subunit vs Placebo: 75.6 (SD=NA) [1 obs]
Mean age Live vs Placebo: NA (SD=NA) [0 obs]

Appendix S13: Funnel Plots to Assess Publication Bias

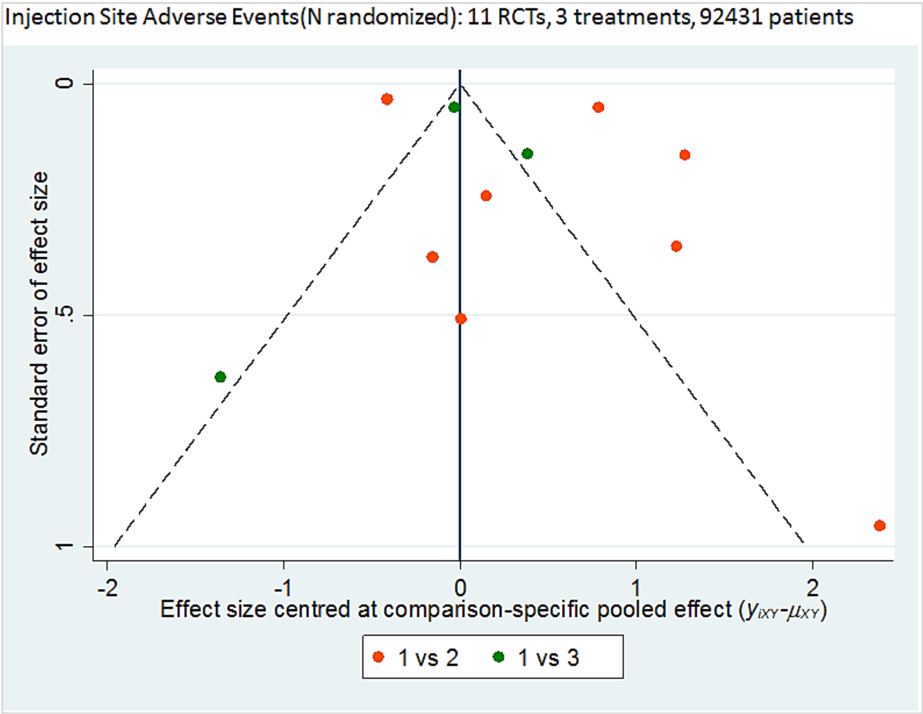

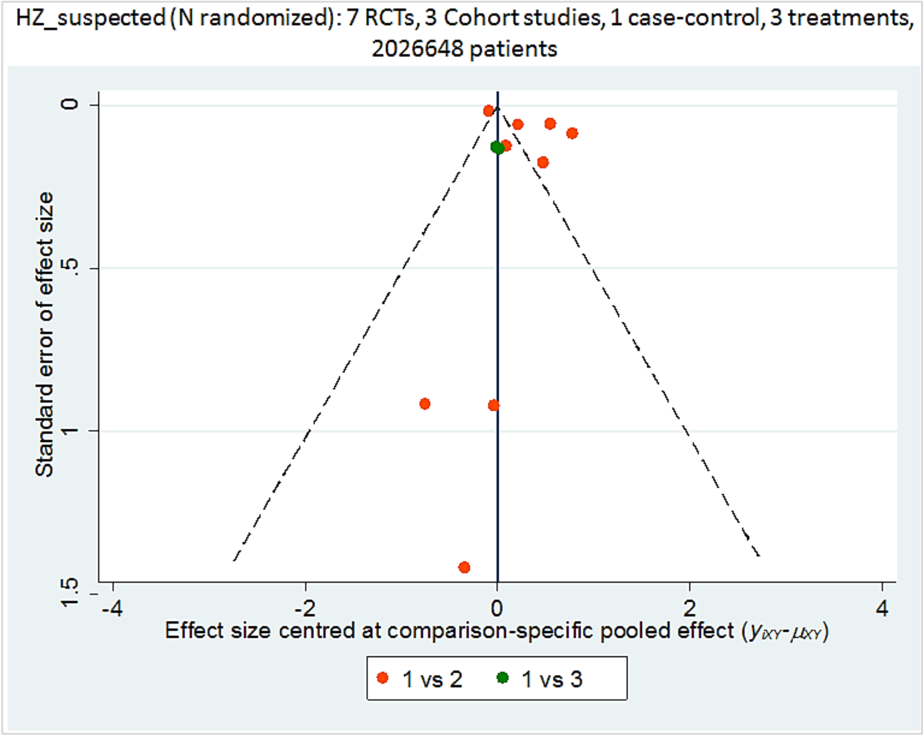

Appendix S14: Additional Results for Physician or Lab Confirmed HZ Cases: Pairwise Meta-Analyses and Network Meta-Analyses

Treatment Comparison	# studies (# patient)	Odds Ratio from direct and indirect comparisons (95% CrI) (95% PrI* *only for indirect comparison*)	MA Risk Ratio from direct comparison (95% CrI)	Risk Ratio from indirect comparison (95% CrI) (95% PrI)	Vaccine Efficacy/ Effectiveness % (95% CrI) ‡
**Effectiveness comparison using all study designs:** 5 RCTs and 1 case-control study with 91,230 patients					
HZ/su vs. ZVL	-	0.15 (0.04-0.45) (0.02-0.80)*	-	0.16 (0.04-0.47) (0.02-0.81)*	85 (55 to 96)*
HZ/su vs. Placebo[8, 19, 20]	2 (29,311)	0.06 (0.02-0.16)*	0.07 (0.03-0.18)*	-	94 (84 to 98)*
ZVL vs. Placebo[24, 26-35]	4 (61,919)	0.43 (0.22-0.98)*	0.45 (0.24-0.98)*	-	57 (2 to 78)*
Common within-network between-study variance			0.19 (0.01-1.91)	0.19 (0.01-1.85)	
**Efficacy comparison of patients with no prior history of HZ vaccination:** 2 RCTs with 29,311 patients					
HZ/su vs. Placebo[8, 19, 20]	2 (29,311)	0.06 (0.01-0.27)*	0.06 (0.01-0.27)*	-	94 (73 to 99)*
Common within-network between-study variance			0.56 (0.01-4.31)		
**Efficacy comparison of patients with no prior history of HZ infection:** 4 RCTs with 52,059 patients					
HZ/su vs. ZVL	-	0.16 (0.01-1.27) (0.00-2.90)	-	0.16 (0.01-1.27) (0.00-2.88)	84 (-27 to 99)
HZ/su vs. Placebo[8, 19, 20]	2 (29,311)	0.06 (0.01-0.28)*	0.06 (0.01-0.28)*	-	94 (72 to 99)*
ZVL vs. Placebo[34, 35]	2 (22,748)	0.39 (0.08-4.61)	0.39 (0.08-4.34)	-	61 (-334 to 92)
Common within-network between-study variance			0.59 (0.02-4.53)	0.60 (0.02-4.54)	
**Efficacy comparison of patients with a history of VZV infection:** 3 RCTs with 61,294 patients					
ZVL vs. Placebo[26-35]	3 (61,294)	0.43 (0.16-1.69)	0.43 (0.16-1.68)	-	57 (-68 to 84)
Common within-network between-study variance			0.26 (0.00-3.51)		
**Efficacy comparison using informative prior (Turner approach)[1]:** 5 RCTs with 90,605 patients					
HZ/su vs. ZVL	-	0.15 (0.04-0.45) (0.03-0.72)*	-	0.15 (0.04-0.45) (0.03-0.72)*	85 (55 to 96)*
HZ/su vs. Placebo[8, 19, 20]	2 (29,311)	0.06 (0.03-0.15)*	0.07 (0.03-0.15)*	-	93 (85 to 97)*
ZVL vs. Placebo[26-35]	3 (61,294)	0.42 (0.20-1.00)	0.42 (0.20-1.00)	-	58 (0 to 80)
Common within-network between-study variance			0.17 (0.02-1.50)	0.17 (0.02-1.45)	
**Sensitivity analysis of RCTs with low risk of bias for random sequence generation:** 4 RCTs with 52,059 patients					
HZ/su vs. ZVL	-	0.16 (0.01-1.24) (0.00-2.53)	-	0.16 (0.01-1.24) (0.00-2.52)	84 (-24 to 99)
HZ/su vs. Placebo[8, 19, 20]	2 (29,311)	0.06 (0.01-0.28)*	0.06 (0.01-0.29)*	-	94 (71 to 99)*

[1] Turner RM, Davey J, Clarke MJ, Thompson SG, Higgins JPT. Predicting the extent of heterogeneity in meta-analysis, using empirical data from the Cochrane Database of Systematic Reviews. International Journal of Epidemiology 2012; 41(3):818–827.

ZVL vs. Placebo[34, 35]	2 (22,748)	0.39 (0.08-4.60)	0.39 (0.08-4.32)	-	61 (-332 to 92)
Common within-network between-study variance			0.61 (0.02-4.50)	0.60 (0.02-4.52)	
**Sensitivity analysis of RCTs with low risk of bias for allocation concealment:** 2 RCTs with 36,339 patients					
HZ/su vs. ZVL	-	-	-	-	-
HZ/su vs. Placebo†[19, 20]	1 (13,900)	0.10 (0.06-0.15)*	0.10 (0.06-0.15)*	-	90 (85 to 94)*
ZVL vs. Placebo†[35]	1 (22,439)	0.30 (0.20-0.44)	0.30 (0.20-0.45)*	-	70 (55 to 80)*
Common within-network between-study variance			0.45 (0.00-4.93)	-	
**Sensitivity analysis of RCTs that only included immunocompetent patients:** 4 RCTs with 90,296 patients					
HZ/su vs. ZVL	-	0.16 (0.03-0.90) (0.01-1.75)*	-	0.16 (0.03-0.90) (0.01-1.73)*	84 (10 to 97)*
HZ/su vs. Placebo[8, 19, 20]	2 (29,311)	0.06 (0.02-0.21)*	0.06 (0.02-0.21)*	-	94 (78 to 98)*
ZVL vs. Placebo[26-33, 35]	2 (60,985)	0.39 (0.11-1.29)	0.40 (0.12-1.28)	-	60 (-28 to 88)
Common within-network between-study variance			0.35 (0.02-3.08)	0.37 (0.02-3.12)	

***** Indicates statistical significance at p < 0.05

† Only one study included in this comparison

‡ For analysis with RCTs only, ***vaccine efficacy*** was computed using the ***risk ratio estimates*** from the NMA or the MA (when no NMA was possible). Analyses that included all study designs (i.e., RCTs, observational studies and quasi experimental studies), ***vaccine effectiveness*** was computed using the ***odds ratio estimates*** from the NMA or MA

Abbreviations: CrI – credible interval; HZ – herpes zoster; HZ/su – adjuvant, recombinant subunit herpes zoster vaccine; MA – meta-analysis; NA – not applicable; NMA – network meta-analysis; PrI – prediction interval; RCT – randomized controlled trials; ZVL – live-attenuated herpes zoster vaccine.

Appendix S15: Additional Results for Suspected HZ Cases: Pairwise Meta-Analyses and Network Meta-Analyses

Treatment Comparison	# studies (# patient)	Odds Ratio from direct and indirect comparisons (95% CrI) (95% PrI* *only for indirect comparison*)	MA Risk Ratio from direct comparison (95% CrI)	Risk Ratio from indirect comparison (95% CrI) (95% PrI)	Vaccine Efficacy/ Effectiveness % (95% CrI) ‡
**Effectiveness comparison using all study designs:** 7 RCTs, 3 cohort studies and 1 case-control study with 2,026,648 patients					
HZ/su vs. ZVL	-	0.43 (0.21-0.80) (0.15-1.14)*	-	0.44 (0.22-0.80) (0.15-1.13)*	57 (20 to 79)*
HZ/su vs. Placebo[8, 19, 20]	2 (29,311)	0.22 (0.13-0.40)*	0.24 (0.13-0.41)*	-	78 (60 to 87)*
ZVL vs. Placebo[9, 10, 21, 22, 24, 26-35, 37-41, 44, 45]	9 (1,997,337)	0.53 (0.39-0.73)*	0.54 (0.41-0.75)*	-	47 (27 to 61)*
Common within-network between-study variance			0.11 (0.04-0.48)	0.11 (0.04-0.48)	
**Network meta-regression for duration of follow-up:** 7 RCTs, 3 cohort studies and 1 case-control study with 2,026,648 patients					
HZ/su vs. ZVL	-	0.44 (0.29-0.62) (0.25-0.75)*	-	0.45 (0.30-0.64) (0.26-0.75)*	56 (38 to 71)*
HZ/su vs. Placebo[8, 19, 20]	2 (29,311)	0.20 (0.15-0.29) (0.12-0.35)*	-	0.22 (0.16-0.30) (0.13-0.36)*	80 (71 to 85)*
ZVL vs. Placebo[9, 10, 21, 22, 24, 26-35, 37-41, 44, 45]	9 (1,997,337)	0.47 (0.39-0.58) (0.31-0.75)*	-	0.48 (0.41-0.59) (0.33-0.76)*	53 (42 to 61)*
Common within-network between-study variance				0.02 (0.00-0.18)	
Regression coefficient				0.99 (0.99-1.00)	
**Sensitivity analysis excluding 3 trials with large standard errors and negative centred effect sizes to explore publication bias:** 4 RCTs, 3 cohort with 2,026,648 patients					
HZ/su vs. ZVL	-	0.44 (0.22-0.87) (0.16-1.30)*	-	0.44 (0.22-0.87) (0.16-1.29)*	56 (13 to 78)*
HZ/su vs. Placebo[8, 19, 20]	2 (29,311)	0.22 (0.12-0.40)*	0.23 (0.13-0.41)	-	77 (59 to 87)*
ZVL vs. Placebo[21, 22, 24, 26-33, 35, 37-41, 44, 45]	6 (1,996,575)	0.51 (0.36-0.71)*	0.52 (0.37-0.72)	-	48 (28 to 63)*
Common within-network between-study variance			0.11 (0.04-0.54)	0.11 (0.04-0.54)	
**Efficacy comparison using informative prior (Turner approach)[1]:** 7 RCTs with 91,840 patients					
HZ/su vs. ZVL	-	0.36 (0.21-0.57) (0.17-0.69)	-	0.37 (0.21-0.58) (0.17-0.69)*	63 (42 to 79)*
HZ/su vs. Placebo[8, 19, 20]	2 (29,311)	0.22 (0.15-0.33)*	0.23 (0.16-0.33)*	-	77 (67 to 84)*
ZVL vs. Placebo[9, 10, 26-35]	5 (62,529)	0.61 (0.46-0.93)*	0.62 (0.47-0.93)*	-	38 (7 to 53)*
Common within-network between-study variance			0.03 (0.00-0.30)	0.03 (0.00-0.30)	

[1] Turner RM, Davey J, Clarke MJ, Thompson SG, Higgins JPT. Predicting the extent of heterogeneity in meta-analysis, using empirical data from the Cochrane Database of Systematic Reviews. International Journal of Epidemiology 2012; 41(3):818–827.

**Sensitivity analysis of RCTs with low risk of bias for random sequence generation:** 4 RCTs with 52,059 patients					
HZ/su vs. ZVL	-	0.35 (0.12-0.99) (0.08-1.47)*	-	0.35 (0.12-0.99) (0.08-1.46)*	65 (1 to 88)*
HZ/su vs. Placebo[8, 19, 20]	2 (29,311)	0.22 (0.11-0.47)*	0.23 (0.12-0.47)*	-	77 (53 to 88)*
ZVL vs. Placebo[34, 35]	2 (22,748)	0.65 (0.29-1.60)	0.66 (0.30-1.57)	-	34 (-57 to 70)
Common within-network between-study variance			0.04 (0.00-1.60)	0.04 (0.00-1.62)	
**Sensitivity analysis of RCTs with low risk of bias for allocation concealment:** 2 RCTs with 36,339 patients					
HZ/su vs. ZVL	-		-	-	-
HZ/su vs. Placebo† [19, 20]	1 (13,900)	0.22 (0.17-0.28)*	0.23 (0.18-0.29)*	-	77 (71 to 82)*
ZVL vs. Placebo†[35]	1 (22,439)	0.65 (0.51-0.81)*	0.65 (0.51-0.82)*	-	35 (18 to 49)*
Common within-network between-study variance			0.45 (0.00-5.20)		
**Sensitivity analysis of RCTs with immunocompetent and potentially immunocompetent patients:** 6 RCTs with 91,531 patients					
HZ/su vs. ZVL	-	0.37 (0.19-0.60) (0.16-0.75)*	-	0.38 (0.19-0.60) (0.16-0.75)*	62 (40 to 81)*
HZ/su vs. Placebo[8, 19, 20]	2 (29,311)	0.23 (0.15-0.34)*	0.23 (0.15-0.35)*	-	77 (65 to 85)*
ZVL vs. Placebo[9, 10, 26-33, 35]	4 (62,220)	0.60 (0.44-0.99)*	0.61 (0.45-0.99)*	-	39 (1 to 55)*
Common within-network between-study variance			0.01 (0.00-0.62)	0.01 (0.00-0.60)	
**Sensitivity analysis of RCTs that only included immunocompetent patients:** 4 RCTs with 90,296 patients					
HZ/su vs. ZVL	-	0.38 (0.20-0.74) (0.15-0.93)*	-	0.38 (0.20-0.74) (0.15-0.93)*	62 (26 to 80)*
HZ/su vs. Placebo[8, 19, 20]	2 (29,311)	0.22 (0.14-0.35)*	0.23 (0.15-0.36)*	-	77 (64 to 85)*
ZVL vs. Placebo[26-33, 35]	2 (60,985)	0.59 (0.39-0.96)*	0.60 (0.39-0.96)*	-	40 (4 to 61)*
Common within-network between-study variance			0.01 (0.00-0.83)	0.01 (0.00-0.84)	

***** Indicates statistical significance at $p < 0.05$

† Only one study included in this comparison

‡ For analysis with RCTs only, ***vaccine efficacy*** was computed using the ***risk ratio estimates*** from the NMA or the MA (when no NMA was possible). Analyses that included all study designs (i.e., RCTs, observational studies and quasi experimental studies), ***vaccine effectiveness*** was computed using the ***odds ratio estimates*** from the NMA or MA

Abbreviations: CrI – credible interval; HZ – herpes zoster; HZ/su – adjuvant, recombinant subunit herpes zoster vaccine; MA – meta-analysis; NA - not applicable; NMA – network meta-analysis; PrI – prediction interval; RCT – randomized controlled trials; ZVL – live-attenuated herpes zoster vaccine.

Appendix S16: Additional Results for HZ Ophthalmicus: Pairwise Meta-Analyses and Network Meta-Analyses

Treatment Comparison	# studies (# patient)	Odds Ratio from direct and indirect comparisons (95% CrI) (95% PrI* *only for indirect comparison*)	MA Risk Ratio from direct comparison (95% CrI)	Risk Ratio from indirect comparison (95% CrI) (95% PrI)	Vaccine Efficacy/ Effectiveness % (95% CrI) ‡
**Sensitivity analysis of RCTs with immunocompetent patients:** 1 RCT with 13,900 patients					
HZ/su vs. Placebo[19, 20]	1 (13,900)	0.17 (0.02-1.38)	0.17 (0.02-1.38)	-	83 (-38 to 98)
Common within-network between-study variance			-		

***** Indicates statistical significance at $p < 0.05$

† Only one study included in this comparison

‡ For analysis with RCTs only, ***vaccine efficacy*** was computed using the ***risk ratio estimates*** from the NMA or the MA (when no NMA was possible). Analyses that included all study designs (i.e., RCTs, observational studies and quasi experimental studies), ***vaccine effectiveness*** was computed using the ***odds ratio estimates*** from the NMA or MA

Abbreviations: CrI – credible interval; HZ/su – adjuvant, recombinant subunit herpes zoster vaccine; MA – meta-analysis; NA – not applicable; NMA – network meta-analysis; PrI – prediction interval; RCT – randomized controlled trials; ZVL – live-attenuated herpes zoster vaccine.

Appendix S17: Additional Results for Post-Herpetic Neuralgia: Pairwise Meta-Analyses and Network Meta-Analyses

Treatment Comparison	# studies (# patient)	Odds Ratio from direct and indirect comparisons (95% CrI) (95% PrI* *only for indirect comparison*)	MA Risk Ratio from direct comparison (95% CrI)	Risk Ratio from indirect comparison (95% CrI) (95% PrI)	Vaccine Efficacy/ Effectiveness % (95% CrI) ‡
**Effectiveness comparison using all study designs:** 2 RCTs, 1 cohort and 1 case-control study with 819,401 patients					
HZ/su vs. ZVL	-	0.30 (0.04-1.94) (0.02-3.22)	-	0.30 (0.04-1.90) (0.02-3.05)	70 (-94 to 96)
HZ/su vs. Placebo† [19, 20]	1 (13,900)	0.13 (0.04-0.34)*	0.13 (0.04-0.35)*	-	87 (66 to 96)*
ZVL vs. Placebo[21, 22, 24, 26-33]	3 (805,501)	0.44 (0.19-1.11)	0.45 (0.19-1.10)	-	56 (-11 to 81)
Common within-network between-study variance			0.20 (0.00-2.43)	0.21 (0.00-2.64)	

***** Indicates statistical significance at $p < 0.05$

† Only one study included in this comparison

‡ For analysis with RCTs only, ***vaccine efficacy*** was computed using the ***risk ratio estimates*** from the NMA or the MA (when no NMA was possible). Analyses that included all study designs (i.e., RCTs, observational studies and quasi experimental studies), ***vaccine effectiveness*** was computed using the ***odds ratio estimates*** from the NMA or MA

Abbreviations: CrI – credible interval; HZ/su – adjuvant, recombinant subunit herpes zoster vaccine; MA – meta-analysis; NA – not applicable; NMA – network meta-analysis; PrI – prediction interval; RCT – randomized controlled trials

Appendix S18: Additional Results for Injection Site Adverse Events: Pairwise Meta-Analyses and Network Meta-Analyses

Treatment Comparison	# studies (# patient)	Odds Ratio from direct and indirect comparisons (95% CrI) (95% PrI* *only for indirect comparison*)	MA Risk Ratio from direct comparison (95% CrI)	Risk Ratio from indirect comparison (95% CrI) (95% PrI)
**Efficacy comparison of patients with no prior history of HZ infection:** 10 RCTs with 53,885 patients				
HZ/su vs. ZVL	-	3.30 (0.96-13.62) (0.39-36.82)	-	1.68 (0.98-2.17) (0.53-2.30)
HZ/su vs. Placebo[1, 8, 19, 20]	3 (29,499)	14.33 (5.08-45.45)*	5.39 (3.37-6.96)*	-
ZVL vs. Placebo[2, 3, 7, 9, 10, 34, 35]	7 (24,386)	4.31 (1.99-8.83)*	3.05 (1.77-4.47)*	-
Common within-network between-study variance			0.65 (0.20-2.24)	0.65 (0.20-2.26)
**Network meta-regression for duration of follow-up:** 11 RCTs with 92,431 patients				
HZ/su vs. ZVL	-	3.57 (0.98-15.96) (0.42-38.40)	-	1.81 (0.99-2.41) (0.54-2.55)
HZ/su vs. Placebo[1, 8, 19, 20]	3 (29,499)	15.04 (3.75-72.20) (1.60-161.68)*	-	5.73 (2.85-7.82) (1.50-8.25)*
ZVL vs. Placebo[2, 3, 7, 9, 10, 26-35]	8 (62,932)	4.20 (1.84-9.24) (0.57-28.84)*	-	3.07 (1.68-4.73) (0.60-6.83)*
Common within-network between-study variance				0.00 (-0.06-0.05)
Regression coefficient				1.89 (1.23-9.18)
**Sensitivity analysis of RCTs with low risk of bias for random sequence generation:** 6 RCTs with 52,383 patients				
HZ/su vs. ZVL	-	2.13 (0.40-15.55) 0.16-38.43)	-	1.53 (0.51-2.55) (0.23-2.72)
HZ/su vs. Placebo[8, 19, 20]	2 (29,311)	9.78 (2.22-42.52)*	5.42 (2.00-8.85)*	-
ZVL vs. Placebo[2, 3, 34, 35]	4 (23,072)	4.50 (1.27-12.51)*	3.41 (1.24-6.09)*	-
Common within-network between-study variance			0.73 (0.13-3.52)	0.73 (0.13-3.41)
**Sensitivity analysis of RCTs with low risk of bias for allocation concealment:** 5 RCTs with 36,851 patients				
HZ/su vs. ZVL	-	2.95 (0.36-46.95) (0.14-144.53)	-	1.66 (0.48-2.44) (0.21-2.49)
HZ/su vs. Placebo[1, 19, 20]	2 (14,088)	16.45 (2.85-124.16)*	6.94 (2.45-10.41)*	-
ZVL vs. Placebo[2, 3, 35]	3 (22,763)	5.64 (1.00-21.92)	4.00 (1.00-7.68)	-
Common within-network between-study variance			1.10 (0.12-4.87)	1.10 (0.12-4.93)
**Sensitivity analysis of RCTs with immunocompetent and potentially immunocompetent patients:** 10 RCTs with 92,122 patients				
HZ/su vs. ZVL	-	3.13 (1.00-12.02) (0.42-28.05)	-	1.69 (1.00-2.22) (0.55-2.37)
HZ/su vs. Placebo[1, 8, 19, 20]	3 (29,499)	14.15 (5.21-43.08)*	5.62 (3.50-7.35)*	-
ZVL vs. Placebo[2, 3, 7, 9, 10, 26-33, 35]	7 (62,623)	4.55 (2.18-8.84)*	3.23 (1.92-4.64)*	-
Common within-network between-study variance			0.58 (0.18-2.12)	0.57 (0.18-2.11)
**Sensitivity analysis of RCTs with immunocompetent patients:** 6 RCTs with 90,620 patients				

HZ/su vs. ZVL	-	1.94 (0.39-11.91) (0.17-27.69)	-	1.49 (0.49-2.66) (0.23-2.91)
HZ/su vs. Placebo[8, 19, 20]	2 (29,311)	9.79 (2.46-38.01)*	5.85 (2.21-9.90)*	-
ZVL vs. Placebo[2, 3, 26-33, 35]	4 (61,309)	4.99 (1.54-12.24)*	3.82 (1.48-6.57)*	-
Common within-network between-study variance			0.59 (0.12-3.06)	0.59 (0.13-3.11)

***** Indicates statistical significance at $p < 0.05$

Abbreviations: CrI – credible interval; HZ/su – adjuvant, recombinant subunit herpes zoster vaccine; MA – meta-analysis; NA – not applicable; NMA - network meta-analysis; PrI - prediction interval; RCT – randomized controlled trials; ZVL – live-attenuated herpes zoster vaccine.

Appendix S19: Additional Results for Systemic Adverse Events: Pairwise Meta-Analyses and Network Meta-Analyses

Treatment Comparison	# studies (# patient)	Odds Ratio from direct and indirect comparisons (95% CrI) (95% PrI* *only for indirect comparison*)	MA Risk Ratio from direct comparison (95% CrI)	Risk Ratio from indirect comparison (95% CrI) (95% PrI)
**Sensitivity analysis of RCTs with low risk of bias for random sequence generation:** 6 RCTs with 52,383 patients				
HZ/su vs. ZVL	-	1.59 (0.23-6.89) (0.11-15.54)	-	1.42 (0.27-3.19) (0.13-4.03)
HZ/su vs. Placebo[8, 19, 20]	2 (29,311)	2.57 (0.68-9.93)	2.13 (0.71-4.54)	-
ZVL vs. Placebo[2, 3, 34, 35]	4 (23,072)	1.65 (0.68-5.98)	1.52 (0.71-3.60)	-
Common within-network between-study variance			0.48 (0.02-3.33)	0.47 (0.02-3.22)
**Sensitivity analysis of RCTs with low risk of bias for allocation concealment:** 5 RCTs with 36,851 patients				
HZ/su vs. ZVL	-	1.53 (0.12-16.56) (0.04-49.16)	-	1.41 (0.14-4.53) (0.05-5.34)
HZ/su vs. Placebo[1, 19, 20]	2 (14,088)	3.59 (0.61-23.16)	2.76 (0.64-6.49)	-
ZVL vs. Placebo[2, 3, 35]	3 (22,763)	2.33 (0.50-15.61)	2.02 (0.53-5.80)	-
Common within-network between-study variance			1.09 (0.16-5.04)	1.10 (0.16-4.90)
**Sensitivity analysis of RCTs with immunocompetent and potentially immunocompetent patients:** 8 RCTs with 90,887 patients				
HZ/su vs. ZVL	-	2.31 (0.61-7.15) (0.30-15.06)	-	1.86 (0.66-3.37) (0.34-4.22)
HZ/su vs. Placebo[1, 8, 19, 20]	3 (29,499)	3.06 (1.37-8.64)*	2.37 (1.30-4.14)*	-
ZVL vs. Placebo[2, 3, 7, 26-33, 35]	5 (61,388)	1.36 (0.72-3.62)	1.29 (0.75-2.64)	-
Common within-network between-study variance			0.34 (0.01-2.21)	0.35 (0.01-2.18)
**Sensitivity analysis of RCTs with immunocompetent patients:** 6 RCTs with 90,620 patients				
HZ/su vs. ZVL	-	1.77 (0.25-7.22) (0.11-15.91)	-	1.60 (0.28-3.88) (0.12-5.19)
HZ/su vs. Placebo[8, 19, 20]	2 (29,311)	2.58 (0.69-9.57)	2.23 (0.71-5.15)	-
ZVL vs. Placebo[2, 3, 26-33, 35]	4 (61,309)	1.48 (0.65-5.68)	1.41 (0.67-3.86)	-
Common within-network between-study variance			0.46 (0.01-3.21)	0.45 (0.01-3.21)

***** Indicates statistical significance at $p < 0.05$

Abbreviations: CrI – credible interval; HZ/su – adjuvant, recombinant subunit herpes zoster vaccine; MA – meta-analysis; NA – not applicable; NMA – network meta-analysis; PrI - prediction interval; RCT – randomized controlled trials; ZVL – live-attenuated herpes zoster vaccine.

Appendix S20: Additional Results for Serious Adverse Events: Pairwise Meta-Analyses and Network Meta-Analyses

Treatment Comparison	# studies (# patient)	Odds Ratio from direct and indirect comparisons (95% CrI) (95% PrI) *(if applicable)*	MA Risk Ratio from direct comparison (95% CrI)	Risk Ratio from indirect comparison (95% CrI) (95% PrI)
**Sensitivity analysis of RCTs with low risk of bias for random sequence generation:** 4 RCTs with 52,119 patients				
HZ/su vs. ZVL	-	0.90 (0.42-2.13) (0.30-3.01)	-	0.90 (0.43-2.05) (0.31-2.80)
HZ/su vs. Placebo[8, 19, 20]	2 (29,311)	0.97 (0.56-1.71)	0.97 (0.58-1.61)	-
ZVL vs. Placebo[34, 35]	2 (22,808)	1.08 (0.57-1.97)	1.07 (0.59-1.82)	-
Common within-network between-study variance			0.02 (0.00-1.15)	0.02 (0.00-1.17)
**Sensitivity analysis of RCTs with low risk of bias for allocation concealment:** 3 RCTs with 48,398 patients				
HZ/su vs. ZVL	-	0.81 (0.16-4.51) (0.10-7.51)	-	0.81 (0.16-4.25) (0.10-6.75)
HZ/su vs. Placebo†[19, 20]	1 (13,900)	0.94 (0.86-1.03)	0.94 (0.87-1.03)	-
ZVL vs. Placebo[25, 35]	2 (34,498)	1.15 (0.44-3.17)	1.14 (0.45-2.76)	-
Common within-network between-study variance			0.08 (0.00-2.68)	0.08 (0.00-2.71)
**Sensitivity analysis of RCTs with immunocompetent and potentially immunocompetent patients:** 7 RCTs with 103,590 patients				
HZ/su vs. ZVL	-	0.89 (0.65-1.18) (0.57-1.34)	-	0.90 (0.65-1.18) (0.57-1.34)
HZ/su vs. Placebo[8, 19, 20]	2 (29,311)	0.97 (0.78-1.21)	0.97 (0.79-1.20)	-
ZVL vs. Placebo[9, 10, 25-33, 35]	5 (74,279)	1.09 (0.91-1.37)	1.08 (0.91-1.34)	-
Common within-network between-study variance			0.00 (0.00-0.16)	0.00 (0.00-0.18)
**Sensitivity analysis of RCTs with immunocompetent patients:** 4 RCTs with 90,356 patients				
HZ/su vs. ZVL	-	0.93 (0.55-1.56) (0.45-1.88)	-	0.93 (0.56-1.55) (0.45-1.85)
HZ/su vs. Placebo[8, 19, 20]	2 (29,311)	0.97 (0.68-1.38)	0.97 (0.69-1.34)	-
ZVL vs. Placebo[26-33, 35]	2 (61,045)	1.05 (0.70-1.60)	1.04 (0.72-1.53)	-
Common within-network between-study variance			0.01 (0.00-0.71)	0.01 (0.00-0.64)

***** Indicates statistical significance at $p < 0.05$
† Only one study included in this comparison

Abbreviations: CrI – credible interval; HZ/su – adjuvant, recombinant subunit herpes zoster vaccine; MA – meta-analysis; NA – not applicable; NMA - network meta-analysis; PrI - prediction interval; RCT – randomized controlled trials; ZVL – live-attenuated herpes zoster vaccine.

Appendix S21: Additional Results for Withdrawal Due to Adverse Events: Pairwise Meta-Analyses and Network Meta-Analyses

Treatment Comparison	# studies (# patient)	Odds Ratio from direct and indirect comparisons (95% CrI) (95% PrI* *only for indirect comparison*)	MA Risk Ratio from direct comparison (95% CrI)	Risk Ratio from indirect comparison (95% CrI) (95% PrI)
**Sensitivity analysis of RCTs with low risk of bias for random sequence generation:** 3 RCTs with 23,018 patients				
ZVL vs. Placebo[3, 34, 35]	3 (23,018)	0.73 (0.22-3.07)	0.73 (0.23-2.98)	-
Common within-network between-study variance			0.30 (0.00-3.69)	
**Sensitivity analysis of RCTs with low risk of bias for allocation concealment:** 4 RCTs with 34,896 patients				
HZ/su vs. ZVL	-	2.44 (0.06-1237.78) (0.04-1580.53)	-	2.40 (0.06-83.34) (0.04-84.56)
HZ/su vs. Placebo†[1]	1 (188)	2.44 (0.12-962.46)	2.43 (0.13-211.63)	-
ZVL vs. Placebo[3, 25, 35]	3 (34,708)	1.01 (0.39-3.58)	1.01 (0.39-3.55)	-
Common within-network between-study variance			0.27 (0.00-3.12)	0.27 (0.00-3.18)
**Sensitivity analysis of RCTs with immunocompetent and potentially immunocompetent patients:** 5 RCTs with 35,369 patients				
HZ/su vs. ZVL	-	2.85 (0.08-2461.24) (0.06-2859.59)	-	2.80 (0.08-101.45) (0.06-102.03)
HZ/su vs. Placebo[1]	1 (188)	2.23 (0.11-1733.10)	2.22 (0.11-228.61)	-
ZVL vs. Placebo[3, 10, 25, 35]	4 (35,181)	0.97 (0.43-2.82)	0.97 (0.43-2.80)	-
Common within-network between-study variance			0.21 (0.00-2.47)	0.21 (0.00-2.80)
**Sensitivity analysis of RCTs with immunocompetent patients:** 3 RCTs with 34,708 patients				
ZVL vs. Placebo[3, 25, 35]	3 (34,708)	1.01 (0.40-3.58)	1.01 (0.40-3.54)	-
Common within-network between-study variance			0.26 (0.00-3.08)	

***** Indicates statistical significance at $p < 0.05$
† Only one study included in this comparison

Abbreviations: CrI – credible interval; HZ/su – adjuvant, recombinant subunit herpes zoster vaccine; MA – meta-analysis; NA – not applicable; NMA - network meta-analysis; PrI - prediction interval; RCT – randomized controlled trials; ZVL – live-attenuated herpes zoster vaccine.

Appendix S22: Additional Results for Death: Pairwise Meta-Analyses and Network Meta-Analyses

Treatment Comparison	# studies (# patient)	Odds Ratio from direct and indirect comparisons (95% CrI) (95% PrI* *only for indirect comparison*)	MA Risk Ratio from direct comparison (95% CrI)	Risk Ratio from indirect comparison (95% CrI) (95% PrI)
**Sensitivity analysis of RCTs with low risk of bias for random sequence generation:** 5 RCTs with 52,173 patients				
HZ/su vs. ZVL	-	1.56 (0.44-4.98) (0.30-7.63)	-	1.54 (0.44-4.71) (0.30-6.96)
HZ/su vs. Placebo[8, 19, 20]	2 (29,311)	0.94 (0.46-1.92)	0.94 (0.46-1.88)	-
ZVL vs. Placebo[2, 34, 35]	3 (22,862)	0.60 (0.24-1.65)	0.61 (0.25-1.63)	-
Common within-network between-study variance			0.04 (0.00-1.82)	0.04 (0.00-1.92)
**Sensitivity analysis of RCTs with low risk of bias for allocation concealment:** 4 RCTs with 48,452 patients				
HZ/su vs. ZVL	-	0.94 (0.08-7.15) (0.04-13.86)	-	0.94 (0.09-6.58) (0.04-11.72)
HZ/su vs. Placebo†[19, 20]	1 (13,900)	0.92 (0.80-1.06)	0.93 (0.81-1.06)	-
ZVL vs. Placebo[2, 25, 35]	3 (34,552)	1.02 (0.32-5.19)	1.01 (0.32-4.83)	-
Common within-network between-study variance			0.42 (0.00-4.00)	0.37 (0.00-3.50)
**Sensitivity analysis of RCTs with immunocompetent and potentially immunocompetent patients:** 6 RCTs with 102,409 patients				
HZ/su vs. ZVL	-	0.95 (0.45-1.97) (0.31-2.71)	-	0.95 (0.46-1.93) (0.32-2.62)
HZ/su vs. Placebo[8, 19, 20]	2 (29.311)	0.94 (0.54-1.62)	0.94 (0.55-1.60)	-
ZVL vs. Placebo[2, 25-33, 35]	4 (73,098)	0.99 (0.63-1.65)	0.99 (0.63-1.63)	-
Common within-network between-study variance			0.03 (0.00-0.97)	0.03 (0.00-0.98)
**Sensitivity analysis of RCTs with immunocompetent patients:** 5 RCTs with 90,410 patients				
HZ/su vs. ZVL	-	1.00 (0.40-2.65) (0.26-3.87)	-	1.00 (0.40-2.54) (0.27-3.60)
HZ/su vs. Placebo[8, 19, 20]	2 (29,311)	0.94 (0.48-1.79)	0.94 (0.49-1.75)	-
ZVL vs. Placebo[2, 26-33, 35]	3 (61,099)	0.94 (0.46-1.89)	0.94 (0.47-1.84)	-
Common within-network between-study variance			0.04 (0.00-1.47)	0.04 (0.00-1.49)

***** Indicates statistical significance at $p < 0.05$
† Only one study included in this comparison

Abbreviations: CrI - credible interval; HZ/su – adjuvant, recombinant subunit herpes zoster vaccine; MA – meta-analysis; NA - not applicable; NMA - network meta-analysis; PrI - prediction interval; RCT - randomized controlled trials; ZVL – live-attenuated herpes zoster vaccine.

Appendix S23: Sensitivity Analysis for Dose-Effects

Treatment Comparison	NMA Risk Ratio (95% CrI) (95% PrI)	Vaccine Efficacy % (95%CrI)
**Suspected HZ Cases**: Analysis excluding one study[6] that administered two shots of low dose ZVL *8 RCTs [4, 5, 8-10, 19, 20, 34, 35] with 53,667 patients and average follow-up of 13 months (range 1 to 44 months)*		
ZVL low dose vs. Placebo	0.59 (0.08-2.10) (0.07-2.92)	41 (-110 to 92)
ZVL standard dose vs. Placebo	0.69 (0.39-1.67) (0.24-2.80)	31 (-67 to 61)
ZVL standard dose vs. ZVL low dose	1.14 (0.40-8.17) (0.30-9.69)	-14 (-717 to 60)
ZVL high dose vs. Placebo	0.72 (0.20-2.51) (0.16-3.45)	28 (-151 to 80)
ZVL high dose vs. ZVL low dose	1.15 (0.38-9.45) (0.27-10.88)	-15 (-845 to 62)
ZVL high dose vs. ZVL standard dose	1.01 (0.28-3.31) (0.22-4.16)	-1 (-231 to 72)
HZ/su standard dose vs. Placebo	0.23 (0.11-0.44) (0.07-0.78)*	77 (56 to 89)*
HZ/su standard dose vs. ZVL low dose	0.39 (0.08-3.03) (0.06-3.66)	61 (-203 to 92)
HZ/su standard dose vs. ZVL standard dose	0.33 (0.10-0.77) (0.07-1.07)*	67 (23 to 90)*
HZ/su standard dose vs. ZVL high dose	0.31 (0.07-1.29) (0.05-1.61)	69 (-29 to 93)
Common within-network between-study variance	0.04 (0.00-1.47)	
Between-dose variance	0.22 (0.00-3.13)	
**Injection Site AE**: Analysis excluding one study[7] that administered two shots of standard dose ZVL 11 RCTs [1-5, 8-10, 19, 20, 34, 35] with 54,119 patients and average follow-up of 24 days (range 5 to 42 days)		
ZVL low dose vs. Placebo	2.63 (0.47-6.50) (0.07-8.43)	NA
ZVL standard dose vs. Placebo	3.03 (0.87-6.17) (0.10-8.42)	NA
ZVL standard dose vs. ZVL low dose	1.05 (0.45-1.86) (0.05-2.12)	NA
ZVL high dose vs. Placebo	2.24 (0.43-5.79) (0.06-8.32)	NA
ZVL high dose vs. ZVL low dose	0.94 (0.23-1.59) (0.03-2.10)	NA
ZVL high dose vs. ZVL standard dose	0.87 (0.17-1.39) (0.03-1.88)	NA
HZ/su standard dose vs. Placebo	1.41 (0.21-5.11) (0.03-8.01)	NA
HZ/su standard dose vs. ZVL low dose	0.60 (0.05-1.87) (0.01-2.10)	NA
HZ/su standard dose vs. ZVL standard dose	0.53 (0.06-1.62) (0.01-1.88)	NA
HZ/su standard dose vs. ZVL high dose	0.64 (0.05-2.91) (0.01-3.68)	NA
Common within-network between-study variance	2.69 (1.20-6.24)	
Between-dose variance	0.28 (0.00-3.56)	
**Systemic AE:** Analysis excluding one study[7] that administered two shots of standard dose ZVL 7 RCTs [1-3, 8, 19, 20, 34, 35] with 52,511 patients and average follow-up of 27 days (range 7 to 42 days)		
ZVL standard dose vs. Placebo	1.81 (0.46-4.56) (0.11-6.38)	NA
ZVL high dose vs. Placebo	1.65 (0.18-5.40) (0.06-6.56)	NA
ZVL high dose vs. ZVL standard dose	0.96 (0.12-2.73) (0.03-3.57)	NA
HZ/su standard dose vs. Placebo	1.05 (0.23-3.24) (0.05-5.83)	NA
HZ/su standard dose vs. ZVL standard dose	0.58 (0.06-2.44) (0.02-3.40)	NA
HZ/su standard dose vs. ZVL high dose	0.58 (0.03-7.74) (0.01-14.63)	NA
Common within-network between-study variance	1.61 (0.50-5.16)	
Between-dose variance	0.37 (0.00-4.40)	

* Indicates statistical significance at $p < 0.05$

Abbreviations: CrI – credible interval; HZ – herpes zoster; HZ/su – adjuvant, recombinant subunit herpes zoster vaccine; NA – not applicable; NMA – network meta-analysis; RCT – randomized controlled trials; ZVL – live-attenuated herpes zoster vaccine.

References

1. Chlibek R, Bayas JM, Collins H, et al. Safety and immunogenicity of an ASO1 -adjuvanted varicella-zoster virus subunit candidate vaccine against herpes zoster in adults ≥50 years of age. *Journal of Infectious Diseases* 2013;208:1953-61. doi: 10.1093/infdis/jit365
2. Hata A, Inoue F, Hamamoto Y, et al. Efficacy and safety of live varicella zoster vaccine in diabetes: a randomized, double-blind, placebo-controlled trial. *Diabetic Medicine* 2016;33:1094-101. doi: 10.1111/dme.13038
3. Vermeulen JN, Lange JMA, Tyring SK, et al. Safety, tolerability, and immunogenicity after 1 and 2 doses of zoster vaccine in healthy adults ≥60 years of age. *Vaccine* 2012;30:904-10. doi: 10.1016/j.vaccine.2011.11.096
4. Beals CR, Railkar RA, Schaeffer AK, et al. Immune response and reactogenicity of intradermal administration versus subcutaneous administration of varicella-zoster virus vaccine: an exploratory, randomised, partly blinded trial. *The Lancet Infectious Diseases* 2016;16:915-22. doi: 10.1016/S1473-3099(16)00133-X
5. Berger R, Trannoy E, Holländer G, et al. A dose-response study of a live attenuated varicella-zoster virus (Oka strain) vaccine administered to adults 55 years of age and older. *The Journal of infectious diseases* 1998;178 Suppl S99-103.
6. Leroux-Roels I, Leroux-Roels G, Clement F, et al. A Phase 1/2 Clinical Trial Evaluating Safety and Immunogenicity of a Varicella Zoster Glycoprotein E Subunit Vaccine Candidate in Young and Older Adults. *Journal of Infectious Diseases* 2012;206:1280-90. doi: 10.1093/infdis/jis497
7. Merck Sharp & Dohme Corp. A Study to Evaluate Immunity to Varicella Zoster Virus After Immunization With V212 Vaccine or Zostavax (V212-003). ClinicalTrialsgov, 2015:1-9.
8. Lal H, Cunningham AL, Godeaux O, et al. Efficacy of an Adjuvanted Herpes Zoster Subunit Vaccine in Older Adults. *New England Journal of Medicine* 2015;372:2087-96. doi: 10.1056/NEJMoa1501184
9. Kerzner B, Murray AV, Cheng E, et al. Safety and immunogenicity profile of the concomitant administration of ZOSTAVAX and inactivated influenza vaccine in adults aged 50 and older. *Journal of the American Geriatrics Society* 2007;55:1499-507. doi: 10.1111/j.1532-5415.2007.01397.x
10. MacIntyre CR, Egerton T, McCaughey M, et al. Concomitant administration of zoster and pneumococcal vaccines in adults ≥60 years old. *Human Vaccines* 2010;6:894-902. doi: 10.4161/hv.6.11.12852
11. Tyring SK, Diaz-Mitoma F, Padget LG, et al. Safety and tolerability of a high-potency zoster vaccine in adults ≥50 years of age. *Vaccine* 2007;25:1877-83. doi: 10.1016/j.vaccine.2006.10.027
12. Diez-Domingo J, Weinke T, Garcia de Lomas J, et al. Comparison of intramuscular and subcutaneous administration of a herpes zoster live-attenuated vaccine in adults aged ≥50 years: A randomised non-inferiority clinical trial. *Vaccine* 2015;33:789-95. doi: 10.1016/j.vaccine.2014.12.024
13. Gilderman LI, Lawless JF, Nolen TM, et al. A double-blind, randomized, controlled, multicenter safety and immunogenicity study of a refrigerator-stable formulation of Zostavax. *Clinical and Vaccine Immunology* 2008;15:314-19. doi: 10.1128/CVI.00310-07
14. GlaxoSmithKline Biologicals. A phase III, randomised, open-label, multicentre, clinical trial to assess the safety and immunogenicity of GSK Biologicals' HZ/su vaccine when administered intramuscularly according to a 0,2-month schedule, a 0,6- month schedule or a 0,12-month schedule in adults aged 50 years or older. EU Clinical Trials, 2016.
15. Vesikari T, Hardt R, Rümke HC, et al. Immunogenicity and safety of a live attenuated shingles (herpes zoster) vaccine (Zostavax ®) in individuals aged ≥ 70 years A

randomized study of a single dose vs . two di ff erent two-dose schedules. *Human Vaccines & Immunotherapeutics* 2013;9:858-64. doi: 10.4161/hv.23412
16. Vink P, Shiramoto M, Ogawa M, et al. Safety and immunogenicity of a Herpes Zoster subunit vaccine in Japanese population aged ≥50 years when administered subcutaneously vs. intramuscularly. *Human Vaccines & Immunotherapeutics* 2017;13:574-78. doi: 10.1080/21645515.2016.1232787
17. Chlibek R, Smetana J, Pauksens K, et al. Safety and immunogenicity of three different formulations of an adjuvanted varicella-zoster virus subunit candidate vaccine in older adults: A phase II, randomized, controlled study. *Vaccine* 2014;32:1745-53. doi: 10.1016/j.vaccine.2014.01.019
18. GSK Clinical Study Register. A phase II, single-blind, randomized, controlled, multicentre vaccination study to evaluate the safety and immune response of the GSK Biologicals Zoster vaccine, gE/AS01B, and to compare 3 doses of gE with AS01B adjuvant in healthy elderly subjects, aged 60 to 69 years and 70 years and above., 2010.
19. Cunningham AL, Lal H, Kovac M, et al. Efficacy of the Herpes Zoster Subunit Vaccine in Adults 70 Years of Age or Older. *New England Journal of Medicine* 2016;375:1019-32. doi: 10.1056/NEJMoa1603800
20. GlaxoSmithKline Biologicals. Ensayo clínico de vacunación fase III, aleatorizado, observador-ciego, controlado con placebo, multicéntrico para evaluar la eficacia profiláctica, seguridad e inmunogenicidad de la vacuna gE/AS01B de GSK Biologicals administrada por vía intramuscular con. EU Clinical Trials, 2016.
21. Langan SM, Smeeth L, Margolis DJ, et al. Herpes Zoster Vaccine Effectiveness against Incident Herpes Zoster and Post-herpetic Neuralgia in an Older US Population: A Cohort Study. *PLoS Medicine* 2013;10 doi: 10.1371/journal.pmed.1001420
22. Langan SM, Thomas SL, Smeeth L, et al. Zoster vaccination is associated with a reduction of zoster in elderly patients with chronic kidney disease. *Nephrology Dialysis Transplantation* 2016;31:2095-98. doi: 10.1093/ndt/gfv432
23. Levin MJ, Schmader KE, Pang L, et al. Cellular and humoral responses to a second dose of herpes zoster vaccine administered 10 years after the first dose among older adults. *Journal of Infectious Diseases* 2016;213:14-22. doi: 10.1093/infdis/jiv480
24. Marin M, Yawn BP, Hales CM, et al. Herpes zoster vaccine effectiveness and manifestations of herpes zoster and associated pain by vaccination status. *Human Vaccines and Immunotherapeutics* 2015;11:1157-64. doi: 10.1080/21645515.2015.1016681
25. Murray AV, Reisinger KS, Kerzner B, et al. Safety and tolerability of zoster vaccine in adults ≥60 years old. *Human Vaccines* 2011;7:1130-36. doi: 10.4161/hv.7.11.17982
26. Oxman MN, Levin MJ, Johnson GR, et al. A Vaccine to Prevent Herpes Zoster and Postherpetic Neuralgia in Older Adults. *New England Journal of Medicine* 2005;352:2271-84. doi: 10.1056/NEJMoa051016
27. Schmader KE, Oxman MN, Levin MJ, et al. Persistence of the efficacy of zoster vaccine in the shingles prevention study and the short-term persistence substudy. *Clinical Infectious Diseases* 2012;55:1320-28. doi: 10.1093/cid/cis638
28. Simberkoff M, Arbeit R, Johnson G, et al. Safety of Herpes Zoster Vaccine in the Shingles Prevention Study. *Annals of internal medicine* 2010;152:545-54. doi: 10.7326/0003-4819-152-9-201005040-00004
29. Oxman Michael N, Levin Myron J. Vaccination against Herpes Zoster and Postherpetic Neuralgia. *The Journal of Infectious Diseases* 2008;197:S228-S36. doi: 10.1086/522159
30. Schmader KE, Johnson GR, Saddier P, et al. Effect of a Zoster Vaccine on Herpes Zoster-Related Interference with Functional Status and Health-Related Quality-of-Life Measures in Older Adults. *Journal of the American Geriatrics Society* 2010;58:1634-41. doi: 10.1111/j.1532-5415.2010.03021.x

31. Ahnn S. FDA Statistical Review and Evaluation Document for the Vaccines and Related Biological Products Advisory Committee (VRBPAC) Protocol 004, 2005:1-14.
32. Levin MJ, Oxman MN, Zhang JH, et al. Varicella-Zoster Virus–Specific Immune Responses in Elderly Recipients of a Herpes Zoster Vaccine. *The Journal of Infectious Diseases* 2008;197:825-35. doi: 10.1086/528696
33. Levin M, Oxman M, Bobrove A, et al. GER-3. Dose-Ranging Safety and Immunogenicity Study of Live Attenuated Varicella-Zoster Virus Vaccine (Oka/Merck) Administered to Adults 60 Years of Age or Older. *Southern Medical Journal* 2005;98:Supplement S55. doi: 0038-4348
34. Russell AF, Parrino J, Fisher CL, et al. Safety, tolerability, and immunogenicity of zoster vaccine in subjects on chronic/maintenance corticosteroids. *Vaccine* 2015;33:3129-34. doi: 10.1016/j.vaccine.2015.04.090
35. Schmader KE, Levin MJ, Gnann JW, et al. Efficacy, safety, and tolerability of herpes zoster vaccine in persons aged 50-59 years. *Clinical Infectious Diseases* 2012;54:922-28. doi: 10.1093/cid/cir970
36. Levin MJ, Schmader KE, Gnann JW, et al. Varicella-zoster virus-specific antibody responses in 50-59-year-old recipients of zoster vaccine. *Journal of Infectious Diseases* 2013;208:1386-90. doi: 10.1093/infdis/jit342
37. Tseng HF, Harpaz R, Luo Y, et al. Declining Effectiveness of Herpes Zoster Vaccine in Adults Aged ≥60 Years. *Journal of Infectious Diseases* 2016;213:1872-75. doi: 10.1093/infdis/jiw047
38. Tseng HF. Herpes Zoster Vaccine in Older Adults and the Risk of Subsequent Herpes Zoster Disease. *JAMA* 2011;305:160. doi: 10.1001/jama.2010.1983
39. Tseng HF, Chi M, Smith N, et al. Herpes zoster vaccine and the incidence of recurrent herpes zoster in an immunocompetent elderly population. *Journal of Infectious Diseases* 2012;206:190-96. doi: 10.1093/infdis/jis334
40. Tseng HF, Tartof S, Harpaz R, et al. Vaccination Against Zoster Remains Effective in Older Adults Who Later Undergo Chemotherapy. *Clinical Infectious Diseases* 2014;59:913-19. doi: 10.1093/cid/ciu498
41. Tseng HF, Luo Y, Shi J, et al. Effectiveness of Herpes Zoster Vaccine in Patients 60 Years and Older With End-stage Renal Disease. *Clinical Infectious Diseases* 2016;62:462-67. doi: 10.1093/cid/civ930
42. Sanofi Pasteur MSD S.N.C. An open-label, randomised, phase 3, comparative, multi-centre study of the immunogenicity and safety of a 1-dose regimen and different 2-dose regimens of a Zoster vaccine (Live), ZOSTAVAX®, in subjects >=70 years of age, 2009:1-13.
43. GlaxoSmithKline. Safety and Immunogenicity Study of GSK Biologicals ' Herpes Zoster Subunit (HZ / su) Vaccine GSK1437173A When Administered Subcutaneously Intramuscularly in Adults Aged ≥ 50 Years. ClinicalTrialsgov, 2017.
44. Zhang J, Xie F, Delzell E, et al. Association Between Vaccination for Herpes Zoster and Risk of Herpes Zoster Infection Among Older Patients With Selected Immune-Mediated Diseases. *JAMA* 2012;308:43-49. doi: 10.1001/jama.2012.7304
45. Zhang J, Delzell E, Xie F, et al. The use, safety, and effectiveness of herpes zoster vaccination in individuals with inflammatory and autoimmune diseases: a longitudinal observational study. *Arthritis Research & Therapy* 2011;13:R174. doi: 10.1186/ar3497
